# METTL1‐m^7^G‐EGFR/EFEMP1 axis promotes the bladder cancer development

**DOI:** 10.1002/ctm2.675

**Published:** 2021-12-22

**Authors:** Xiaoling Ying, Bixia Liu, Zusen Yuan, Yapeng Huang, Cong Chen, Xu Jiang, Haiqing Zhang, Defeng Qi, Shulan Yang, Shuibin Lin, Junhang Luo, Weidong Ji

**Affiliations:** ^1^ Center for Translational Medicine The First Affiliated Hospital Sun Yat‐sen University Guangzhou 510080 China; ^2^ Department of Urology The First Affiliated Hospital Sun Yat‐sen University Guangzhou 510080 China; ^3^ Department of Urology Minimally Invasive Surgery center Guangdong Key Laboratory of Urology The First Affiliated Hospital of Guangzhou Medical University Guangzhou 510230 China

**Keywords:** bladder cancer, EFEMP1, EGFR, METTL1, N7‐methylguanosine (m7G)

## Abstract

**Background:**

The posttranscriptional modifications of transfer RNA (tRNA) are critical for all aspects of the tRNA function and have been implicated in the tumourigenesis and progression of many human cancers. By contrast, the biological functions of methyltransferase‐like 1 (METTL1)‐regulated m^7^G tRNA modification in bladder cancer (BC) remain obscure.

**Results:**

In this research, we show that METTL1 was highly expressed in BC, and its level was correlated with poor patient prognosis. Silencing METTL1 suppresses the proliferation, migration and invasion of BC cells in vitro and in vivo. Multi‐omics analysis reveals that METTL1‐mediated m^7^G tRNA modification altered expression of certain target genes, including EGFR/EFEMP1. Mechanistically, METTL1 regulates the translation of EGFR/EFEMP1 via modifying certain tRNAs. Furthermore, forced expression of EGFR/EFEMP1 partially rescues the effect of METTL1 deletion on BC cells.

**Conclusions:**

Our findings demonstrate the oncogenic role of METTL1 and the pathological significance of the METTL1‐m^7^G‐EGFR/EFEMP1 axis in the BC development, thus providing potential therapeutic targets for the BC treatment.

## Graphical abstract

Our findings demonstrate the oncogenic role of METTL1 and the pathological significance of the METTL1‐m7G‐EGFR/EFEMP1 axis in BC development, thus providing potential therapeutic targets for the BC treatment.

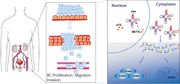

## 1 INTRODUCTION

Bladder cancer (BC) is the ninth most common malignancy globally, with approximately 430,000 cases newly diagnosed every year.^1^ Urothelial carcinoma (UC) accounts for approximately 90% of bladder tumours. The prevalence of BC is three times higher in men than in women.^1^ At initial diagnosis, about 70% of all cases are determined as non‐muscle‐invasive bladder cancer (NMIBC), while 30% are muscle‐invasive bladder cancer (MIBC), which frequently exhibits rapid progression, distant metastasis, and a consequently poor prognosis.^2^ Further, while recurrence is observed in 50%–70% of NMIBC cases, 10%–20% progress into MIBC. Unfortunately, despite surgery and adjuvant therapies, 50% of MIBC patients die from metastasis^3^ and there has been little progress in the treatment of BC over the past two decades.^4^ Thus, it is imperative to elucidate the molecular mechanisms of BC and identify potential therapeutic targets for the improved BC treatment.

The recent progress in epitranscriptomics has been allowed for the identification of over 170 types of post‐transcriptional ribonucleic acid (RNA) modifications of eukaryotic RNA.^5^, ^6^ Among these, *N*6‐methyladenosine (m^6^A) is the most common and abundant modification of mRNA.^7^, ^8^, ^9^, ^10^ Transfer RNAs (tRNAs) undergo various modifications with major effects on their structure, folding, and stability, as well as on protein expression.^11^, ^12^, ^13^, ^14^, ^15^ The dysregulation of tRNA modifications is associated with various cancers.^16^ However, the underlying molecular mechanisms are not well understood. The modification of *N*7‐methylguanosine at position 46 (m^7^G46) of tRNAs is observed in a wide range of prokaryotes, some archaea, and eukaryotes.^13^, ^17^ In yeast, Trm8 and Trm82 form a complex, which carries out m^7^G modification, resulting in the degradation of tRNAVal/AAC via the rapid tRNA degradation (RTD) pathway and thus contributing to a temperature‐sensitive phenotype.^18^ In humans, the loss of WDR4/METTL1‐mediated m^7^G modification is associated with primordial dwarfism (PD).^19^ tRNA modifications and modifying enzymes have also been shown to play crucial roles in the tumour development, suggestive of their potential as therapeutic targets.

Methyltransferase‐like protein 1 (METTL1) forms a complex with WDR4, responsible for tRNA m^7^G modification in humans. Several studies have also reported METTL1‐mediated m^7^G modification in mammalian miRNAs and internal mRNAs.^20^, ^21^, ^22^ However, a recent study did not detect m^7^G modifications in miRNAs using mutational profiling sequencing (m^7^G‐MaP‐seq).^23^ Previous research has shown that the abnormal level of METTL1 is strongly associated with the tumourigenesis and progression of cancer.^21^, ^24^, ^25^ Okamoto et al.^24^ reported that METTL1 inhibition enhanced the sensitivity of HeLa cells to 5‐fluorouracil (5‐FU). In contrast, METTL1 was suggested to suppress lung cancer.^21^ Moreover, METTL1 overexpression promoted the proliferation and migration of hepatocellular carcinoma via the phosphatase and tensin homolog deleted on chromosome ten (PTEN) signalling pathway,^25^ suggesting that METTL1's function may vary between different tumour types. METTL1 deficiency was recently described to cause ribosome pausing at m^7^G‐tRNA‐dependent codons and decrease the translation of cell cycle genes.^26^ However, the role and molecular mechanism of METTL1 in BC have not yet been reported.

In this study, we show that METTL1 was upregulated in BC and was correlated with poor prognosis. Functional assays indicted that METTL1 promoted BC progression both in vitro and in vivo. Mechanistically, METTL1 specifically affected the translation of epidermal growth factor receptor (EGFR)/EFEMP1 (EGF‐containing fibulin extracellular matrix protein 1); by modifying certain tRNAs during the tRNA–mRNA codon recognition. Our findings provide the novel therapeutic targets for BC as well as insights into its molecular pathophysiology.

## 2 MATERIALS AND METHODS

### 2.1 Clinical specimens

Human BC and adjacent noncancerous tissues were obtained from 174 patients who receive radical cystectomy or bladder biopsy from February 2010 to September 2017 at the First Affiliated Hospital of Sun Yat‐sen University (Guangzhou, China) or the First Affiliated Hospital of Guangzhou Medical University (Guangzhou, China). Baseline patient's information is provided in Table S1. The BC tissue microarray (TMA) (HBlaU066Su01, HBlaU079Su01) was obtained from Shanghai Outdo Biotech Company (Shanghai, China). Ethical approval was obtained from the Research Ethics Committee of each institute. Consent form was acquired from all patients prior to the study.

### 2.2 Plasmid construction and siRNA

METTL1 knockout clustered regularly interspaced short palindromic repeats (CRISPR) system singleguide ribonucleic acid (sgRNAs) were designed with the CRISPR online tool (http://chopchop.cbu.uib.no/). Double‐stranded sgRNA oligonucleotides were annealed and inserted into the BsmBI‐digested lentiCRISPRv2. Full‐length EGFR and EFEMP1 complementary DNA (cDNA) were synthesized by Suzhou GENEWIZ Co. Full‐length METTL1 cDNA was cloned from human umbilical endothelial cell (HUVEC) cDNA via polymerase chain reaction (PCR) with primers containing the BamHI and NotI sites. The purified PCR product and pLEX‐MCS vector (Addgene_128044) were digested with BamHI and NotI at 37°C for 3 h. The PCR product was linked into the digested vector. The siRNAs targeting EGFR/EFEMP1 were selected using design tool (https://rnaidesigner.thermofisher.com/rnaiexpress/). The sequences of sgRNA, siRNA and primers are presented in Table S2.

### 2.3 Cell culture

Human immortalized uroepithelial cells (SV‐HUC‐1) and 293T cells (ECACC Cat# 12022001) were purchased from the American Type Culture Collection (Manassas, VA, USA), while human BC cell lines (5637, J82, T24, and UM‐UC‐3) were obtained from the Shanghai Institute of Cell Biology, Chinese Academy of Sciences (Shanghai, China). Human BC cell lines T24 and 5637 were maintained in RPMI‐1640 medium (Gibco, Cat. #: C11875500BT). J82, UM‐UC‐3, and HEK‐293 T cells were grown in DMEM (Gibco, Cat. #: C11885500BT). SV‐HUC‐1 cells were cultured in F‐12K medium (Fisher Scientific, Cat. #: 21127‐022). All culture media were supplemented with 10% fetal bovine serum (FBS, Gibco, Cat. #: 10270106) and 100 U/ml penicillin/streptomycin (Life Technologies, Cat. #: 15140‐122). All cells were cultured at in a 37°C humidified incubator with 5% CO_2_. All cell lines were free of mycoplasma infection.

### 2.4 Construction of stable cell lines

To generate stable METTL1 knockout cell lines, sgRNAs and lentivirus‐packing expression plasmids (psPAX2 and PMD2G) were co‐transfected into 293T cells with Lipofectamine® 3000 reagent (Invitrogen, Cat. #: L3000‐015). To construct stable overexpression cell lines, the target plasmids and packaging vectors pCMV‐dR8.2‐dvpr and PLP‐VSVG were transfected into 293T cells at a 1:1:0.5 ratio. Supernatants containing the virus were collected after 48 h and used to infect BC and SV‐HUC‐1 cells for 24 h together using Polybrene (8 μg/ml, Sigma). Stably transfected cells were treated with 2 μg/ml puromycin for 5 days. Then, stable cell lines were confirmed via western blot.

### 2.5 Immunohistochemistry and immunofluorescence staining

Immunohistochemistry (IHC) and immunofluorescence (IF) staining were carried out as described previously.^27^ Paraffin‐embedded tissue sections were obtained from the Department of Pathology. And the histological examination of tumour tissues was performed by a pathologist. For IHC, human tissues were maintained at 65°C for 30 min and then rehydrated in a gradient of ethanol. Endogenous peroxidase was inhibited using 3% H_2_O_2_, followed by antigen retrieval. Thereafter, the samples were incubated with primary antibodies overnight after blocking using 5% bovine serum albumin (BSA) for 30 min. A semiquantitative scoring system was used to assess IHC staining, as previously described.^28^ For the IF assay, cells were fixed with 4% formaldehyde and then blocked using 1% BSA for 1 h at the room temperature. The cells were then incubated with first antibodies overnight, followed by incubation with fluorescent secondary antibodies. The antibodies used in IHC and IF assays are shown in Table S3.

### 2.6 RNA isolation and qRT‐PCR

Total RNA was isolated from cells with TRIzol reagent (ambion, Cat. #: 15596018) following the manufacturer's protocol. Extracted RNA was reverse transcribed into cDNA with the kit for quantitative real time polymerase chain reaction (q‐PCR) (Vazyme Biotech, Cat. #: R323‐01). Total RNA was first treated using the rtStra™ tRF and tiRNA Pretreatment Kit (Arraystar, Cat. #: AS‐FS‐005), and cDNA was synthesized for tRNA analysis using the rtStra™ First‐Strand cDNA Synthesis Kit (3’ and 5’ adapters) (Arraystar, Cat. #: AS‐FS‐003‐02). qPCR was conducted with the qPCR Mix (Vazyme Biotech, Cat. #: Q711‐02). β‐Actin and U6 were employed as endogenous controls for normalization, and the relative mRNA expression was calculated via the 2 ^−△△CT^ method. The oligonucleotide sequences for qRT‐PCR are presented in Table S4.

### 2.7 Northwestern blot and western blot

Northwestern and western blotting were conducted as described.^26^ For the northwestern blotting of tRNAs, 2 μg total RNA was denatured with 2X TBE loading buffer (sangon Biotech, Cat. #: E307DA0004) at 80°C for 2 min and added into 10% TBE‐UREA gel. Following electrophoresis, the RNAs were then transferred onto a Hybond‐N positively charged nylon membrane. The membrane was then crosslinked with UV and blotted with an anti‐m^7^G antibody (MBL International Cat# RN017M). The m^7^G signals were detected as previously described.^27^

### 2.8 Proliferation assay

Stably transfected cells (2000–5000 cells/well) were added into 96‐well plates and grown for 0–5 days at 37°C in a humidified incubator with 5% CO_2_. Cell proliferation was quantified using a CellTiter 96 AQueous One Solution Cell Proliferation Assay kit (Promega, Cat. #: G3588). Briefly, 3‐(4,5‐dimethylthiazol‐2‐yl)‐5‐(3‐carboxymethoxyphenyl)‐2‐(4‐sulfophenyl)‐2H‐tetrazolium, inner salt (MTS) solution was added to cells (20 μl/well) and incubated at 37°C for 2 h. The absorbance at 490 nm was recorded by a Synergy microplate reader (BioTEK, USA).

### 2.9 Migration assay

Cell migration assays were conducted using an IncuCyte 96‐Well Real‐Time Cell Migration System (Essen Bioscience, USA). Cells (1 × 10^5^/well) were seeded into 96‐well Essen ImageLock plates (Essen Bioscience, USA) and grown to almost 100% confluence. A 96‐pin Wound Maker was used to create homogeneous scratch wounds through a confluent cell monolayer. The plate was washed with cold phosphate‐buffered saline (PBS) and scanned every hour for 24 h. The data were assessed based on the relative wound density, and images were obtained via phase‐contrast imaging. Data were analysed using GraphPad Prism software.

### 2.10 Transwell invasion and transendothelial invasion assays

Transwell Matrigel invasion assays and transendothelial invasion experiments were conducted with 24‐well transwell inserts containing a polycarbonate membrane (8‐μm pore size; Corning). For the transendothelial invasion experiment, 1 × 10^5^ HUVEC cells were seeded into transwell plates before being coated with Corning Matrigel matrix (Corning, Cat. #: 354234) for 24 h. Then, the cells were stained with CellTracker™ CM‐DiI Dye (Invitrogen, Cat. #: C7000) following the manufacturer's instructions and added into the upper chambers. 500 μl mediumwith 20% FBS was then added into the lower chambers. The plates were maintained at 37°C for 15 h. The invading cells were then fixed with 4% paraformaldehyde and stained with 0.1% crystal violet. Cells were then counted using a Zeiss Axio Imager.Z2 microscope.

### 2.11 Xenograft tumour models

All animal and experimental procedures were approved by our Institutional Ethics Committee for clinical research and animal research of Sun Yat‐sen University ([2017]257). Five‐week‐old BALB/cJNju‐Foxn1nu/Nju nude mice were obtained from the Nanjing Biomedical Research Institute of Nanjing University. For subcutaneous implantation, 5 × 10^6^ cells were subcutaneously injected into nude mice. Tumour formation and growth were measured weekly, and the mice were euthanized after 4 weeks. Tumours were weighed and fixed in 4% paraformaldehyde for subsequent IHC assays. Tumour volume was measured using the formula: tumour volume = length × width^2^ × 0.52.

The Tg (Flil: EGFP [enhanced green fluorescent protein]) transgenic zebrafish were used as in vivo zebrafish model systems. Zebrafish were raised and maintained in standard zebrafish units at Sun Yat‐sen University. Tumour cells were stained with CellTracker™ CM‐DiI Dye (Invitrogen, Cat. #: C7000) following the manufacturer's protocol and then injected into the yolk sac or vessels of 2‐day‐old zebrafish embryos using stereoscopic microinjection. Zebrafish embryos were observed daily for 7 days after injection. The number of cells transferred to the tail or perforating blood vessels of zebrafish embryos in each group was recorded every day. Pictures of the zebrafish were taken with a Zeiss Lumar 12 stereomicroscope and light‐sheet microscopy (Carl Zeiss, Germany).

### 2.12 Polysome profiling

Polysome profiling was conducted as described.^28^ First, cells were treated with cycloheximide (CHX, 100 μg/ml, sigma, Cat. #: C7698) for 15 min at 37°C and washed with cold PBS. Then, cells were lysed in polysome lysis buffer on ice for 10 min and then centrifuged at 12 000× rpm for 15 min at 4°C. Supernatants were added into a 10%–50% sucrose gradient and ultracentrifuged at 36,000 rpm and 4°C for 2.5 h using a SW41 rotor (Beckman Coulter, USA). Fractions were collected for qRT‐PCR using the BR‐188 Density Gradient Fractionation System (Brandel, USA).

### 2.13 Identification and quantification of newly synthesized proteins

This experiment was performed as previously described.^29^ To deplete endogenous methionine, the cells were maintained for 30 min with methionine‐free medium followed by incubation with 50 μM AHA (Invitrogen, Cat. #: C10102) for 45 min. AHA‐labelled cells were washed twice using PBS and collected with extraction buffer. The lysates were treated with 40 μM biotin‐FLAG‐alkyne by a Click‐iT Protein Reaction Buffer Kit (Invitrogen, Cat. #: C10276) following the kit protocol. Total proteins were extracted using methanol/chloroform and resuspended in 50 mM Tris, pH 7.5, and 0.01% sodium dodecyl sulfate (SDS). The tagged proteins (1 mg) were incubated with 50 μl streptavidin magnetic beads (Dynabeads M‐280 Streptavidin, Invitrogen, Cat. #: 11206D) for 5 h at room temperature and washed with PBS containing 0.5% SDS. The levels of newly synthesized protein were detected via the western blot.

### 2.14 m^7^G tRNA reduction and cleavage sequencing (TRAC‐seq)

TRAC‐seq was carried out as described.^30^ Briefly, small RNAs were extracted from total RNA using the miRNA isolation kit (Invitrogen, Cat. #: AM1561) and incubated with wild‐type and mutant AlkB proteins to discard methylation from RNAs. Half of the Alkb‐treated small RNAs were saved as input, while the remaining were treated with 0.1M NaBH4 for 30 min on ice and protected from light, then precipitated with ethanol. The NaBH4‐treated RNAs were then resuspended in an aniline–acetate solution. The RNAs were avoided light and incubated for 2 h at the room temperature. Then, RNAs were purified and employed for cDNA library construction, followed by sequencing on illumina next500. The TRAC‐seq data were analysed as previously described.^26^

### 2.15 Ribosome profiling sequencing (Ribo‐seq)

Ribo‐seq was implemented with the TruSeq Ribo Profile kit (Illumina). Briefly, cells were pulsed with 100 μg/ml CHX (Sigma) for 15 min. Ribosome‐protected fragments were produced via nuclease digestion and purified using the RNA clean and concentrator‐25 kit (Zymo Research, Cat. #: R1017). rRNA was removed with a Ribo‐Zero Gold kit (Illumina, Cat. #: MRZG12324). These purified ribosome protected fragment (RPF) and fragmented input RNA samples were then subjected to library construction with the NEBNext Multiple Small RNA Library Prep Set for Illumina (New England Biolabs, Cat. #: E7300L) and sequenced on an illumina HiSeq X Ten. Ribo‐seq data were analysed as previously described.^26^

### 2.16 RNA‐seq analysis

Transcriptome sequencing was performed by Magigene Co. (Guangdong, China). Total RNA was extracted using TRIzol reagent. RNA concentration and quality were assessed by a NanoDrop 2000 system (Thermo Fisher Scientific). RNA integrity and gDNA contamination were tested via denaturing agarose gel electrophoresis. To remove ribosomal RNA, RNA was further purified using a RiboMinus Eukaryote Kit (ThermoFisher, Cat. #: A1083708). Sequencing library concentration was measured using an Agilent 2100 Bioanalyzer and a DNA 1000 chip kit (Agilent, CA, USA). Prior to the cluster generation, the libraries were adjusted to 10 nM. Finally, the samples were analysed on a HiSeq 2000 system (Illumina, San Diego, CA, USA). RNA‐seq read counts were normalized and differential gene expression were analysed using DESeq2.

### 2.17 Tandem mass tag quantitative proteomic analysis

Proteomic analysis was performed using Jingjie PTM BioLab (Hangzhou, China). Briefly, proteins were extracted from the BC cell samples. The protein solution was then reduced using 5 mM dithiothreitol and alkylated with 11 mM iodoacetamide. Finally, trypsin was added to the diluted protein samples for digestion. After trypsin digestion, peptides were labelled with a tandem mass tag (TMT)/iTRAQ kit following the manufacturer's instruction. The labelled peptides were then analysed by LC–MS/MS. Proteins were further classified via gene ontology (GO) and Kyoto Encyclopaedia of Genes and Genomes (KEGG) pathway analysis. Protein domain enrichment analysis was conducted with the InterPro database, while enrichment‐based clustering was performed using GO or KEGG data with a corrected *P*‐value < 0.05.

### 2.18 Statistical analysis

All statistical analyses were conducted by GraphPad Prism 8.0 and SPSS. The experiments in our study were independently repeated at least three times. These results are presented as the mean ± scanning electron microscopies (SEM) and were analysed via a two‐sided unpaired Student's *t*‐test or repeated measure analysis of variance using GraphPad Prism. Association between METTL1 expression and survival in BC were assessed with Kaplan–Meier survival curve and Log‐rank test using GraphPad Prism. The receiver‐operator characteristic (ROC) curve analysis was performed with R software. *P*‐values for every result are indicated on figures, and *P* ≤ 0.05 was deemed statistically significant and labelled with * (*P < 0.05, **P < 0.01, ***P < 0.001, ****P < 0.0001).

### 2.19 Data availability

The RNA‐Seq data obtained in the present research are stored in the National Center for Biotechnology Information (NCBI) Sequence Read Archive under Accession PRJNA733588. TRAC‐seq data generated in this research are stored in the NCBI Sequence Read Archive under Accession PRJNA738633.The Ribo‐Seq data are stored into NCBI Sequence Read Archive with accession PRJNA738263. The proteomics data have been stored in the PRIDE archive with accession PXD026559.

## 3 RESULTS

### 3.1 METTL1 expression is upregulated in bladder cancer and correlates with poor prognosis

**FIGURE 1:**
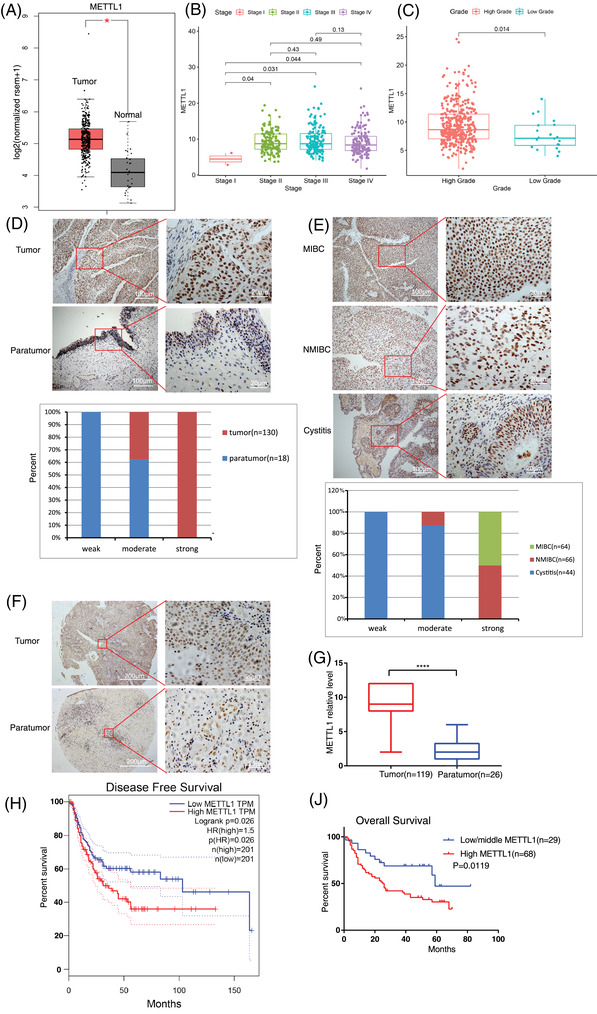
METTL1 is upregulated in bladder cancer (BC) and associated with poor prognosis. (A) Comparison of METTL1 mRNA expression between tumour and normal tissue in TCGA dataset (*n* = 404 in tumour and *n* = 28 in normal, *P < 0.05). (B) Expression of METTL1 in BC based on individual cancer stages in TCGA dataset(*n *= 412). (C) Comparison of METTL1 mRNA expression between high grade and low grade in BC in TCGA dataset (*n* = 392 in high grade tumour and *n* = 20 in low grade tumour, **P = 0.014). (D) Representative immunohistochemistry (IHC) staining images of METTL1 expression in tumour tissues and matched paratumour tissues in patient sample. The nether panel showed histogram of METTL1 expression in tumour and paratumour. Scale bars: (left panel) 100 μm, (right panel) 20 μm. (E) Upper panel: representative IHC staining images of METTL1 expression in muscle‐invasive bladder cancer (MIBC), non‐muscle‐invasive bladder cancer (NMIBC) and cystitis; lower panel: histogram of METTL1 expression in cystitis and bladder cancer tissues. Scale bars: (left panel) 100 μm, (right panel) 20 μm. (F) Representative IHC staining images of METTL1 expression in tumour tissues and matched paratumour tissues in BC tissue microarray (TMA). Scale bars: (left panel) 200 μm, (right panel) 20 μm. (G) Statistical analysis of METTL1 expression between paratumour tissues (*n *= 26) and tumour tissues (*n *= 119) in BC TMA (****P < 0.0001). (H) The Kaplan–Meier survival curve of the disease‐free survival in TCGA database (*P = 0.026). (I) The Kaplan–Meier survival curve of the overall survival between 97 patients with high or low/middle METTL1 expression in BC TMA (*P = 0.0119)

To explore the potential function of the m^7^G tRNA modification in BC, we first assessed m^7^G tRNA methyltransferase METTL1 mRNA expression in BC data from the TCGA (the cancer genome atlas) database. The expression of METTL1 in BC tumour tissues was remarkably higher than in normal tissues (Figure 1A). In addition, METTL1 expression in stages II–IV was higher than in stage I (Figure 1B). Moreover, METTL1 was upregulated in high‐grade BC cancer tissues relative to low‐grade tumours (Figure 1C), indicating that METTL1 may act as an oncogene in BC. Since METTL1 requires its co‐factor WDR4 for its methyltransferase activity, the expression of WDR4 was also analysed in BC tissues using TCGA dataset. The result showed that WDR4 mRNA levels have no significant difference between BC tissues and normal tissues (*P* > 0.05) (Figure S1A). Then, WDR4 protein levels were examined in four BC cell lines (T24, UM‐UC‐3, 5637 and J82) and uroepithelial SV‐HUC‐1 cells using Western blot analysis. There was no obvious difference between BC cell lines and uroepithelial SV‐HUC‐1 cells (*P* > 0.05) (Figure S1B,C). We further determined METTL1 protein expression in human bladder tumour specimens using IHC. Our results were in agreement with those obtained from the TCGA data. IHC revealed high METTL1 protein expression in tumour samples and low expression in paratumour tissue (Figure 1D,F,G). Weak METTL1 expression was observed in the majority of human cystitis tissue samples analysed. METTL1 was upregulated in NMIBC samples relative to cystitis tissue. Moreover, an even greater upregulation of METTL1 was observed in MIBC samples (Figure 1E). Kaplan–Meier survival analysis demonstrated that patients with higher METTL1 expression had significantly poorer disease‐free survival (Figure 1H,I). We further performed ROC curve analysis. The results showed that the 1‐, 3‐, 5‐ years area under the ROC curve (AUC) values of METTL1 were both larger than 0.5 in TCGA data and BC TMA, and 1‐ year area AUC of METTL1 were both over 0.58 (Figure S1D,E) indicating that METTL1 has some predictive significance. Taken together, our results indicated that METTL1 was strikingly upregulated in BC and that METTL1 expression was associated with BC progression, highlighting the potential clinical significance of METTL1 in BC.

### 3.2 METTL1 promotes bladder cancer tumourigenesis in vitro and in vivo

**FIGURE 2:**
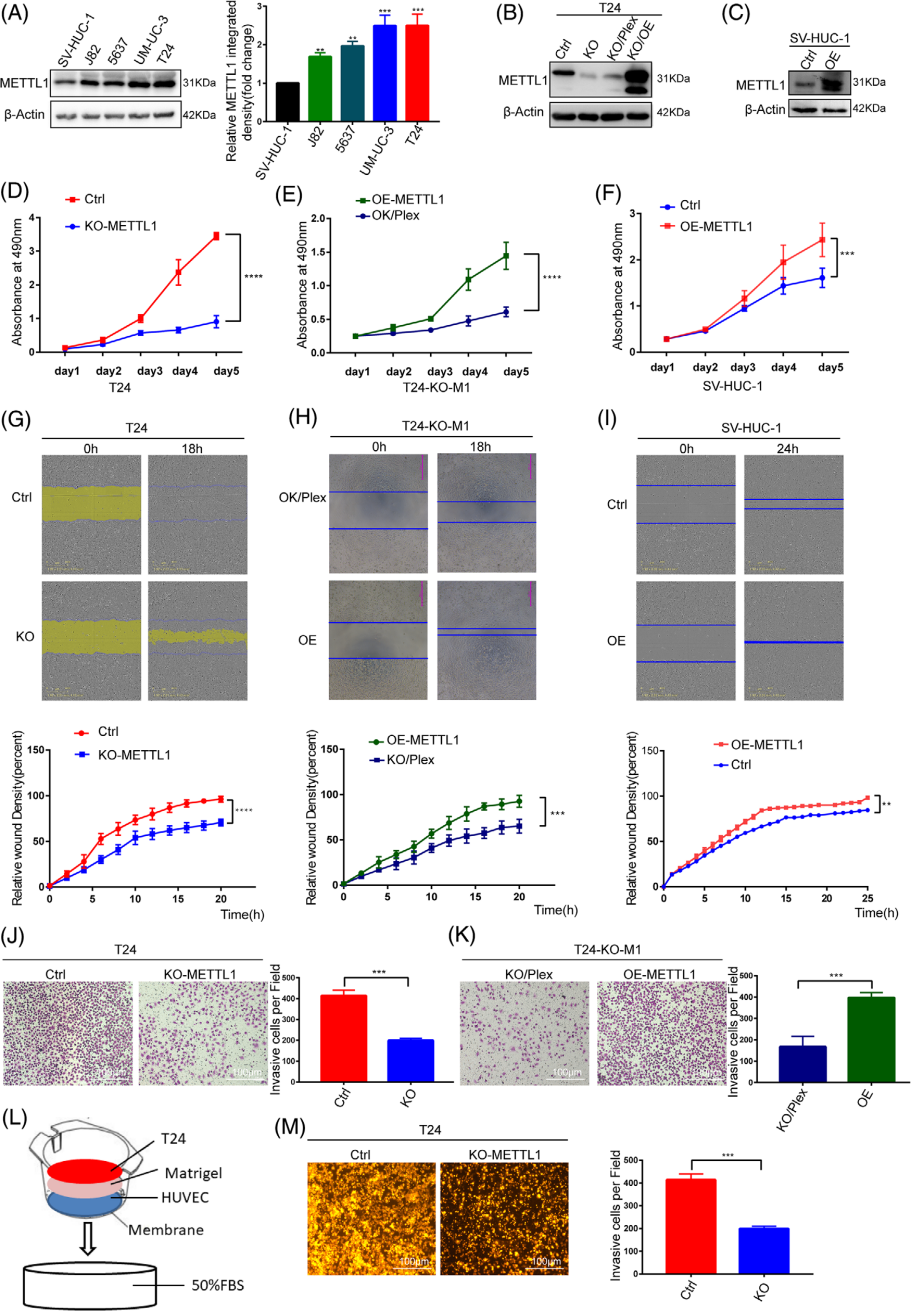
METTL1 promotes tumourigenesis in vitro. (A) METTL1 protein expression in normal and bladder cancer (BC) cell lines. The right panel showed the quantification of Western blot data. (B) Western blot analysis of METTL1 expression in METTL1 stable knockout or rescue cells. (C) Western blot analysis of METTL1 expression in METTL1 stable overexpression cells. (D) MTS assay showed that METTL1 knockout could reduce the viability of BC cells (****P  <  0.0001). (E) MTS assay showed that overexpression METTL1 after knockout could rescue the viability of BC cells (****P  <  0.0001). (F) MTS assay showed that overexpressed METTL1 increased cellular proliferation in SV‐HUC‐1 cells (***P  <  0.001). (G,H) Cell scratch migration assay revealed that the migration capacity was impaired by METTL1 downregulation and rescue by overexpression METTL1 after knockout (***P  <  0.001, ****P  <  0.0001). (I) Migration ability of SV‐HUC‐1 cell was enhanced by METTL1 upregulation (**P  <  0.01). (J) Transwell invasion assays showed that METTL1 knockdown could decrease the invasive abilities of BC cells (***P  <  0.001). (K) Invasive ability of METTL1 knockout cells was rescued by METTL1 overexpression (***P  <  0.001). (L) Schematic presentation of in vitro transendothelial invasion assay. (M) Depletion of METTL1 resulted in decreases in cell invasion ability of BC cells in transendothelial invasion assay (***P  <  0.001). Scale bars: (J, K, M) 100 μm

To further study the function of METTL1 in BC development and progression, we determined METTL1 expression in BC cell lines. METTL1 protein expression was remarkably higher in BC cell lines than in human uroepithelial SV‐HUC‐1 cells (Figure 2A). We chose the two BC cell lines (T24, UM‐UC‐3) with highest expression to construct METTL1 stable knockout BC cells using CRISPR/Cas9 technology (Figures 2B, S2B). Three sgRNAs (sgRNA1, sgRNA2 and sgRNA3) were designed to target the human METTL1 gene (Figure S2A). SgRNA1 was used to generate stable knockout cell lines. METTL1 was overexpressed following knockout to create stable rescue cell lines (Figure 2B). A METTL1‐overexpressing SV‐HUC‐1 cell line was also constructed (Figure 2C). Compared with empty vector control groups, METTL1 knockout BC cells exhibited remarkably decreased cell growth (Figures 2D, S2C). Moreover, wound‐healing and transwell invasion assays demonstrated that METTL1 knockout dramatically inhibited the migratory and invasive abilities of BC cells (Figures 2G,J, S2D,E). To confirm these findings, we also performed a transendothelial invasion assay, in which cancer cells invade through a monolayer of endothelial cells (Figure 2L). The results indicated that METTL1 deficiency obviously inhibited the ability of BC cells to transmigrate across endothelial cell monolayers (Figure 2M). When METTL1 was overexpressed in the SV‐HUC‐1 cell line, cell proliferation and migration were significantly enhanced relative to empty vector controls (Figure 2F,I). In addition, the compromised proliferation, migration and invasion observed in METTL1‐depleted T24 cells were rescued by overexpression of METTL1, confirming its role in promoting cancer progression (Figure 2E,H,K).

**FIGURE 3:**
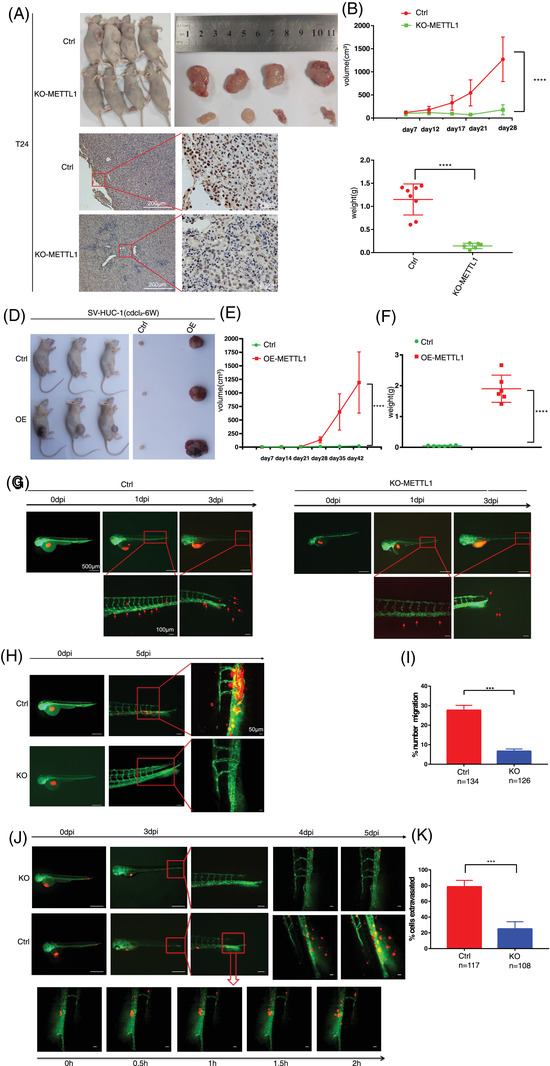
METTL1 promotes tumourigenesis in vivo. (A) Subcutaneous tumour model showed that tumours generation in mice injecting with METTL1 knockout cells had significantly smaller volume and weight than those injected with control cells. The nether panel showed representative immunohistochemistry (IHC) staining images of METTL1 in subcutaneous xenograft tumours (*n* = 8 in each group) Scale bars: (left panel) 200 μm, (right panel) 20 μm. (B) Growth curves of subcutaneous xenograft tumours (****P  <  0.0001). (C) Weight of subcutaneous xenograft tumours (****P  <  0.0001). (D) Subcutaneous tumour model showed that tumours generation in mice injecting with METTL1 overexpression cells had significantly greater volume and weight than those injected with control cells (*n* = 6 in each group). (E) Growth curves of subcutaneous xenograft tumours (****P  <  0.0001). (F) Weight of subcutaneous xenograft tumours (****P  <  0.0001). (G) Zebrafish yolk sac injected with CM‐RED cells showed distant metastases. Red shows bladder cancer (BC) cells, green shows zebrafish blood vessels. Red arrowhead indicates cells that migrate to tail. (H) Tumour proliferation in the tail and neovascularization were found in the T24 control group. (I) Data were expressed as the percent of cells that migrate to tail (number of cells that migrate to tail /total number of cells that injected in yolk sac *100). (*n *= 134 in T24 control group, *n *= 126 in KO‐METTL1 group, ***P  <  0.001). (J) Zebrafish vascular injected with CM‐RED cells showed perforating blood vessel. Red shows BC cells, green shows zebrafish blood vessels. Red arrowhead indicates cells that perforating blood vessel. (K) Data were expressed as the percent of cells that perforating blood vessel (number of cells that perforating blood vessel /total number of cells that injected in vascular *100). (*n* = 117 in T24 control group, *n *= 108 in KO‐METTL1 group, ***P  <  0.001). Scale bars: (G, H, J, left panel) 500 μm, (G, H, J, middle panel) 100 μm, (G, H, J, right panel) 50 μm

To further verify the significance of METTL1 in tumourigenesis in vivo, we employed nude mice as xenograft tumour models. METTL1‐depleted and empty vector control T24 cells were implanted into immunodeficient mice via subcutaneous inoculation, and tumour size was determined at different time points. Mice injected with METTL1‐depleted T24 cells developed tumours of smaller volume and lower tumour weight (Figure 3A,C), which grew significantly slower than their control cell‐derived counterparts (Figure 3B). IHC analysis confirmed that METTL1‐depleted tumours had reduced METTL1 protein expression (Figure 3A). In addition, METTL1 expression significantly enhanced BC growth in METTL1‐overexpressing SV‐HUC‐1 cells treated with chemical carcinogen CdCl_2_ for 6 weeks (Figure 3D–F). We further validated the effects of METTL1 on tumourigenesis and metastasis using transgenic zebrafish xenografts (Flil:EGFP), whose vasculature expresses EGFP. CM‐RED METTL1‐depleted and empty vector control T24 cells were injected into the zebrafish yolk sac or vasculature. The number of cells to migrate from the yolk sac to the tail was recorded every day after injection. We observed that some cells migrated to the tail on the third day after injection (Figure 3G). The number of METTL1‐depleted cells that migrated to tails was smaller than observed for control cells (Figure 3G,I). Moreover, massive tumour proliferation and neovascularization were observed in the tails of control group zebrafish, but not in zebrafish from the METTL1‐depleted group, on the 5th day after injection (Figure 3H). Fewer cells perforating vessels were observed in zebrafish injected with METTL1‐depleted cells (Figure 3J,K). Taken together, the in vivo data confirmed that METTL1 knockout inhibited tumourigenesis and progression in both the xenograft mouse model and the zebrafish tumour model. Overall, METTL1 functional studies indicated that it is indispensable for the growth and progression of BC.

### 3.3 METTL1‐mediated m^7^G tRNA modification regulates tRNA levels

**FIGURE 4:**
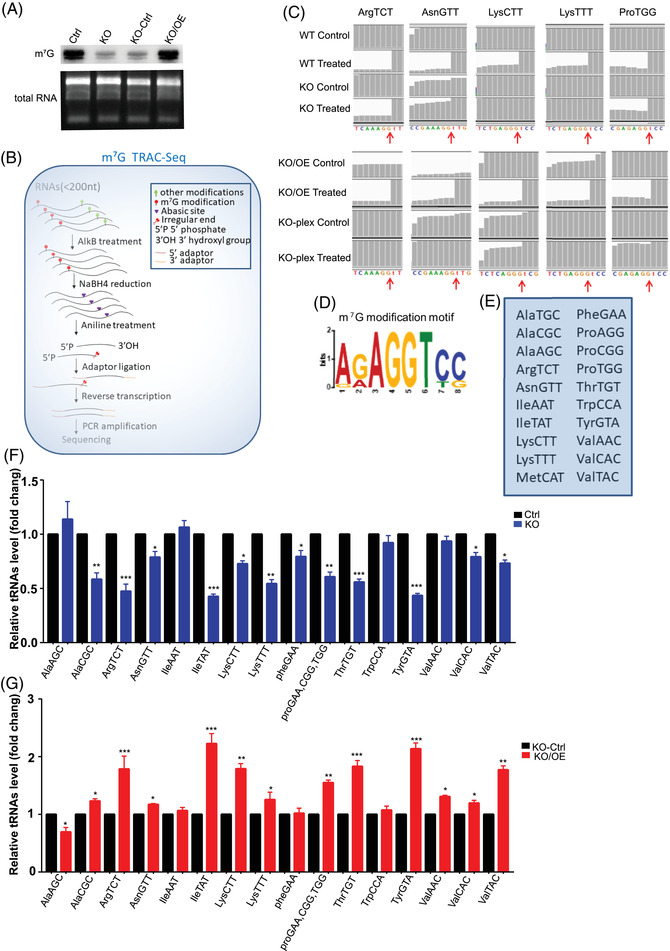
METTL1 mediated m^7^G transfer RNA (tRNA) modification regulates tRNA level. (A) Anti‐m^7^G Northwestern blot of m^7^G modifications. (B) Schematic of tRNA reduction and cleavage sequencing (TRAC‐seq). (C) Representative graphs of reads alignment to identified tRNAs in the integrative genomics viewer (IGV) utilizing m^7^G TRAC‐seq data. (D) Sequence motif in the m^7^G sites indicted by TRAC‐seq in bladder cancer (BC) cells. (E) TRAC‐seq indicted 20 m^7^G‐modified tRNAs in BC cells. (F,G) Analysis of abundance of tRNAs with m^7^G modification using RT‐qPCR (*P < 0.05, **P < 0.01, ***P  <  0.001, ****P  <  0.0001, two‐tailed)

As METTL1 has been confirmed as a tRNA m^7^G methyltransferase,^26^ we assessed the correlation between METTL1‐mediated m^7^G and tRNA levels in order to explore the modification's molecular mechanism in BC. m^7^G levels were determined via northwestern blotting under different METTL1 expression conditions. The m^7^G level was decreased in METTL1‐depleted cells and upregulated under METTL1 overexpression (Figure 4A). We then explored the m^7^G‐modified tRNAs in BC cells via TRAC‐seq (Figure 4B).^30^ Consequently, we identified an m^7^G modification motif of “RAGGU” (Figure 4D) and 20 tRNAs containing the m^7^G modification at the tRNA variable loop (Figure 4E). m^7^G TRAC‐seq data revealed the site‐specific cleavage of m^7^G tRNAs (Figure 4C). To explore the association between tRNA modification levels and tRNA abundance, we detected tRNA abundance with or without m^7^G modifications via RT‐qPCR. Fourteen out of the 20 m^7^G‐modified tRNAs were downregulated in METTL1 knockout cells, and their expression could be rescued via METTL1 overexpression (Figure 4F,G). In contrast, the levels of non‐m^7^G tRNAs were not significantly affected by METTL1 knockout (Figure S3). Overall, our results indicated that METTL1‐mediated m^7^G modification regulates tRNA levels.

### 3.4 METTL1‐mediated m^7^G tRNA modifications regulate mRNA translation through codon recognition in bladder cancer

**FIGURE 5:**
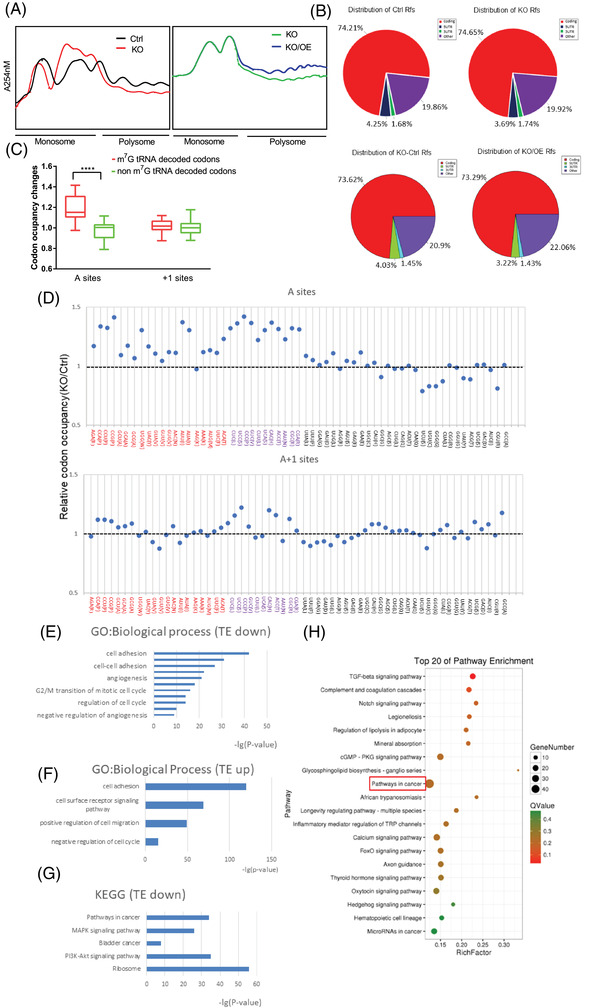
METTL1 mediated m^7^G transfer RNA (tRNA) modification regulates mRNA translation through codon recognition in bladder cancer (BC). (#) Polysome profiling of the METTL1 KO, empty vector control T24 cells, METTL1 expression after knocking out METTL1 T24 cells and METTL1 KO control T24 cells. (B) Distribution of ribosome protected fragment (RPF). (C) Ribosome occupancy changes at A and A+1 sites for codons decoded by m^7^G tRNAs and non m^7^G tRNAs (****P < 0.0001). (D) Ribosome occupancy at individual codon at A and A+1 sites in METTL1 knockout and control cells. The codons were separated into three groups based on the modification of their tRNAs: codons decoded by m^7^G tRNAs (red); codons decoded by non‐m^7^G tRNAs by wobble effect which were not detected levels of their corresponding tRNAs (purple); and codons decoded by non‐m^7^G tRNAs (black). (E) Gene ontology (GO) analysis of function enrichment in biological process using the translation efficiency (TE) downregulated genes upon METTL1 KO. (F) GO analysis of function enrichment in the biological process using the TE upregulated genes upon overexpression METTL1 after METTL1 KO. (G) Kyoto Encyclopaedia of Genes and Genomes (KEGG) pathway enrichment analysis of the TE‐downregulated genes upon METTL1 KO. (H) KEGG pathway enrichment analysis of TE‐upregulated genes upon overexpression METTL1 after METTL1 KO

Since tRNA is mainly involved in protein synthesis, it is plausible to speculate that the abnormal tRNA expression may affect mRNA translation. Polysome profiling was performed to explore the function of m^7^G tRNA modifications in the regulation of mRNA translation. METTL1 depletion caused a reduction of the polyribosome peak, while METTL1 overexpression in METTL1 knockout cells recovered the polyribosome peak (Figure 5A). These results suggested that METTL1‐mediated m^7^G tRNA modifications regulate the mRNA translation in BC cells. To further elucidate the regulatory effect of METTL1‐mediated m^7^G tRNA modification on translation, we performed Ribo‐seq in four groups: METTL1 knockout T24 cells and empty vector control T24 cells; METTL1 rescue T24 cells and METTL1 knockout empty vector control T24 cells. As expected, ribosome‐protected fragments were mainly located within the coding sequence region (Figure 5B). We compared ribosome occupancy between METTL1 knockout cells and controls to find that the knockout of METTL1 caused a significant increase in ribosome pausing at m^7^G‐tRNA‐dependent codons within A sites, while there was little change in ribosome occupancy at the control sites (A+1 sites) (Figure 5C,D), suggesting that the METTL1‐mediated m^7^G tRNA modification is necessary for codon recognition during mRNA translation. We also found a close association between decreased m^7^G‐tRNA abundance and increased A‐site occupancy (Figures 4F,5C). Taken together, these results indicated that METTL1‐mediated m^7^G tRNA modification regulates mRNA translation through codon recognition.

GO enrichment analysis revealed that translation efficiency (TE)‐affected genes decreased upon METTL1 knockout were involved in cell adhesion, cell cycle and angiogenesis (Figure 5E). TE‐upregulated genes under METTL1 rescue in knockout cells were enriched in cell adhesion, cell cycle and cell migration (Figure 5F). These findings suggested a function of METTL1‐mediated m7G tRNA methylation in the regulation of cell proliferation, migration and invasion. We further performed KEGG pathway enrichment analysis of TE‐downregulated genes in METTL1 knockout cells and TE‐upregulated genes under METTL1 overexpression in METTL1 knockout cells. KEGG enrichment analysis revealed that the TE‐affected genes were closely associated with cancer‐related pathways, such as the PI3K‐Akt, MAPK and BC signalling pathways (Figure 5G, H). Overall, our data suggested that METTL1‐mediated m^7^G tRNA methylation was implicated in regulating the translation of BC‐related genes.

### 3.5 METTL1‐mediated m^7^G tRNA modification upregulates EGFR/EFEMP1 protein expression

**FIGURE 6:**
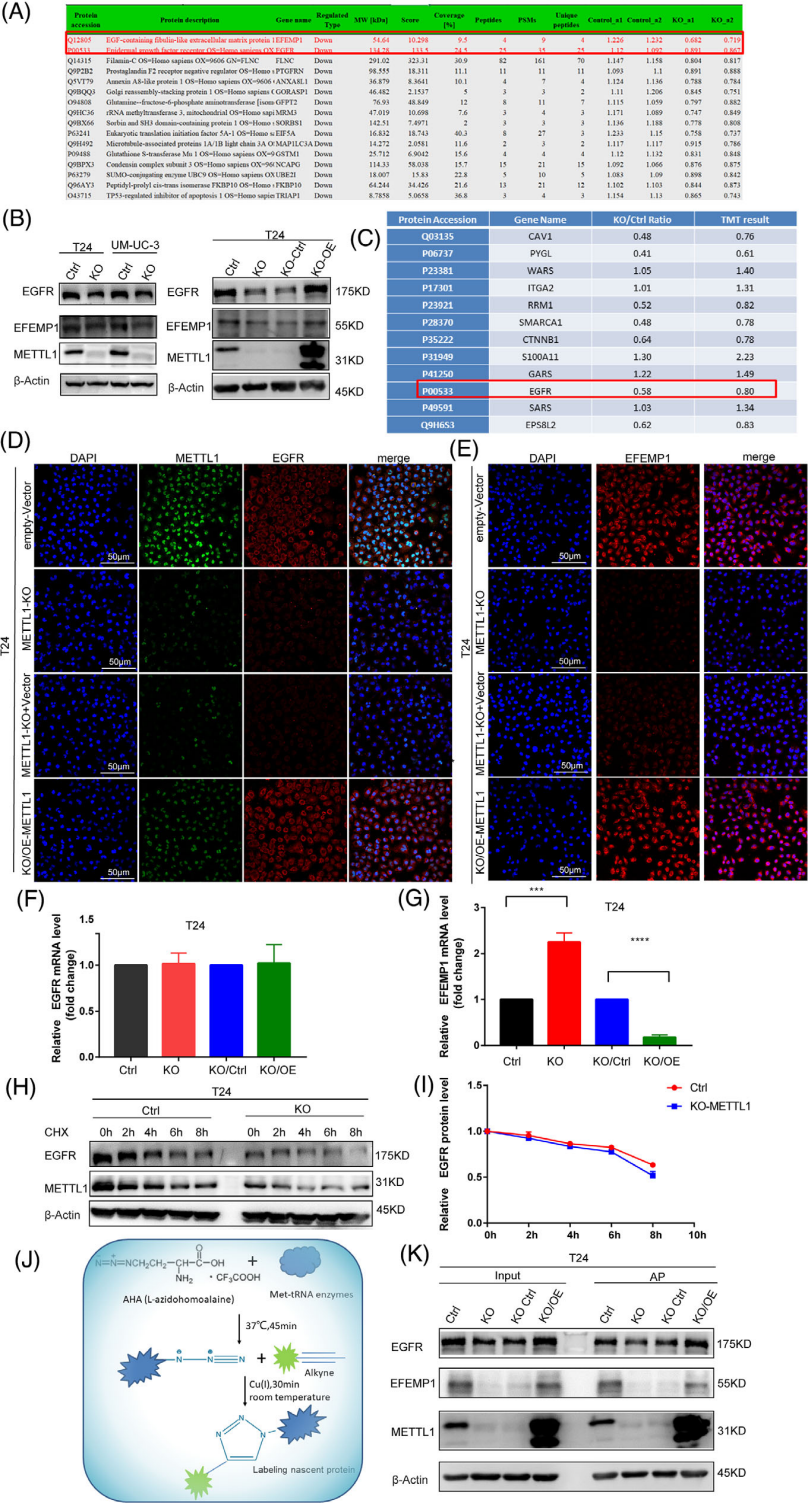
METTL1 mediated m^7^G transfer RNA (tRNA) modification upregulates epidermal growth factor receptor (EGFR)/EFEMP1 protein expression. (A) Significantly downregulated proteins upon METTL1 KO from protein seq data. (B) Left panel: western blotting analysis of protein expression of EGFR and EFEMP1 upon METTL1 KO in T24 and UM‐UC‐3 cells. Right panel: western blotting analysis of protein expression of EGFR and EFEMP1 upon METTL1 KO or overexpression METTL1 after knockout METTL1 in T24 cells. (C) Protein levels by tandem mass tag (TMT) quantitative upon METTL KO. (D,E) Immunofluorescence analyses of EGFR and EFEMP1 (scale bar: 50 μm). (F,G) Q‐PCR analyses of EGFR and EFEMP1 mRNA level (***P < 0.001, ****P < 0.0001). (H,I) Western blotting analysis of EGFR and statistical chart of EGFR relative expression level at the indicated time points after treatment with cycloheximide (CHX). (J) Chematic of detecting the nascent protein synthesis experiment. (K) Western blotting analyse the nascent protein synthesis levels of EGFR and EFEMP1 upon METTL1 KO or overexpression METTL1 after knockout METTL1 in T24 cells. Scale bars: (D,E) 50 μm

To further identify the downstream targets affected by METTL1‐mediated m^7^G tRNA modifications, we performed transcriptome sequencing and proteomic analysis in four groups: METTL1 knockout T24 cells and empty vector control T24 cells; METTL1 rescue T24 cells and METTL1 knockout empty vector control T24 cells. Transcription profiling analysis revealed 384 differentially expressed genes (DEGs) with at least a two‐fold change upon METTL1 knockout. These included 222 downregulated genes and 162 upregulated genes (Figure S4A). A total of 1 674 DEGs were determined upon overexpression of METTL1 after knockout, of which 764 genes were upregulated, and 910 were downregulated (Figure S4B). GO analysis indicated that METTL1 knockout and rescue DEGs were involved in cancer‐related biological processes, including cell adhesion, cell cycle and angiogenesis (Figure S4C–F). Further, KEGG enrichment analysis revealed that the DEGs were closely related to cancer‐related pathways, such as the PI3K‐Akt, MAPK and BC signalling pathways (Figure S4G). These results were consistent with the Ribo‐seq results, further supporting a significant role of METTL1 in the bladder tumour development. A total of 6 526 proteins were identified, and the levels 5 880 were quantified. Compared with the empty vector control group, 193 proteins were upregulated, and 130 proteins were downregulated in the METTL1 knockout group (Figure S5A,C). Compared with the METTL1 knockout control group, 136 proteins were increased, and 39 proteins were decreased in METTL1 recuse cells (Figure S5B,D). To further explore protein functions, differentially expressed proteins (DEPs) were analysed via GO enrichment (Figure S5E,I), clusters of orthologous groups of proteins classification (Figure S5F,J), subcellular localization (Figure S5G,K), and protein‐domain enrichment (Figure S5H,L). The EGFR and EFEMP1 were significantly downregulated in METTL1 knockout cells (Figure 6A). The EGFR is a key receptor involved in signalling pathways driving tumour progression. The PI3K/AKT pathway is downstream of the EGFR and can be activated by the EGFR–ligand interaction to drive cell proliferation, survival and invasion.^31^, ^32^ KEGG analysis revealed that DEGs were enriched in the PI3K‐Akt pathway (Figures 5G,H, S4G). Protein‐domain analysis revealed an enrichment of EGF domains in the METTL1 knockout group (Figure S5H,L). EFEMP1 (EGF‐containing fibulin‐like extracellular matrix protein 1) binds EGFR, resulting in EGFR autophosphorylation and the activation of downstream signalling pathways. A previous study revealed that EFEMP1 bound EGFR, activating the MAPK and Akt pathways to promote tumour growth in pancreatic carcinoma cells.^33^ Therefore, we focused on the association between METTL1‐mediated m^7^G tRNA modification and EGFR/EFEMP1 in the subsequent experiments.

To further confirm whether METTL1‐mediated m^7^G tRNA modification upregulates EGFR/EFEMP1 protein expression, we performed western blotting and IF assays. Western blotting revealed that the protein levels of EGFR and EFEMP1 decreased after METTL1 knockout in T24 and UM‐UC‐3 cells (Figures 6B, S6A,B). In addition, METTL1 overexpression in METTL1‐depleted T24 cells restored the expression of both EGFR and EFEMP1 (Figures 6B, S6C,D). The protein level of EGFR was quantitated using TMT mass spectrometry, and the result was in agreement with western blot data (Figure 6C). We further validated these findings via IF. METTL1 knockout decreased the protein levels of EGFR/EFEMP1, which were increased after METTL1 rescue in T24 cells (Figure 6D,E). qPCR analysis of EGFR mRNA expression revealed no significant differences between METTL1‐depleted and METTL1‐competent cells (Figure 6F). The mRNA level of EFEMP1 was downregulated upon METTL1 knockout and restored upon METTL1 rescue (Figure 6G). These data suggested that the altered EGFR/EFEMP1 protein expression was not due to changes in transcription. To elucidate the mechanism underlying the METTL1‐mediated regulation of EGFR/EFEMP1, protein stability and *de novo* synthesized proteins were evaluated. The depletion of METTL1 did not affect EGFR protein stability (Figure 6H,I). Further, knockout of METTL1 caused a decrease in EGFR/EFEMP1 *de novo* protein synthesis. Newly produced EGFR/EFEMP1 increased under METTL1 overexpression after knockout (Figure 6J,K). In conclusion, these results confirmed that METTL1‐mediated m^7^G tRNA modification upregulated EGFR/EFEMP1 protein expression.

### 3.6 METTL1‐mediated m^7^G tRNA modification regulates the translation of EGFR/EFEMP1

**FIGURE 7:**
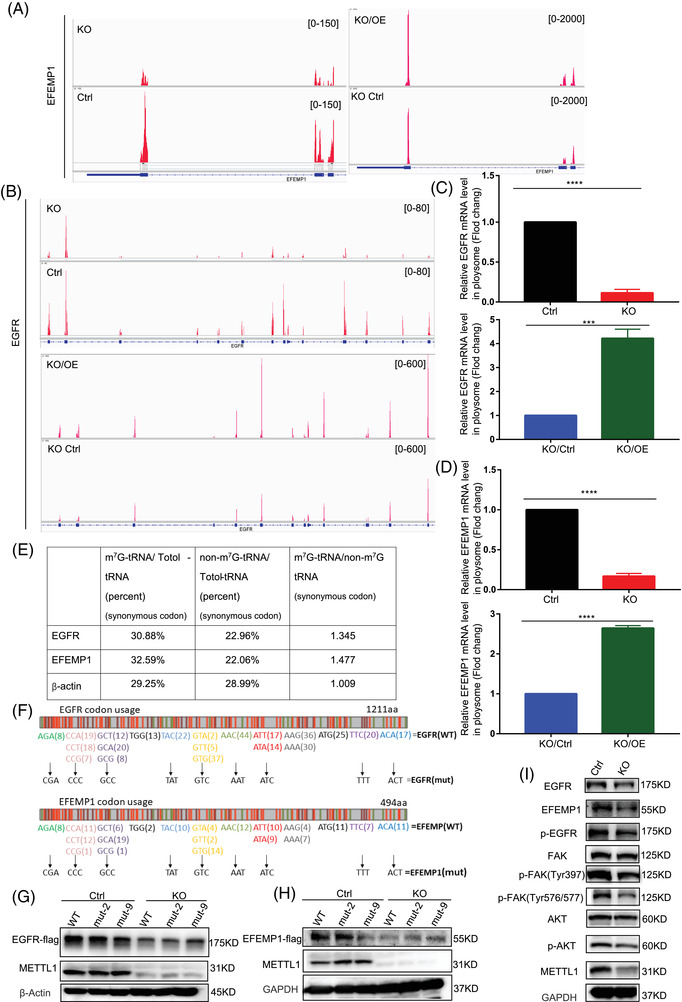
METTL1 mediated m^7^G transfer RNA (tRNA) modification regulates the translation of epidermal growth factor receptor (EGFR)/EFEMP1. (A,B) Integrative genome viewer (IGV) images showing translated mRNA expressions of EGFR and EFEMP1 from Ribo‐seq. (C,D) qRT‐PCR analysis of input mRNA expression and polyribosome bound mRNA levels of EGFR and EFEMP1 genes (***P < 0.001, ****P < 0.0001). (E) Codon analysis of m^7^G tRNA decoded codons of EGFR and EFEMP1. (F) Representation of EGFR and EFEMP1 mRNAs. (G,H) Western blotting analysis of protein expression of EGFR and EFEMP1 in T24 control and METTL1‐KO cells overexpressing EGFR/EFEMP1 WT–Flag, EGFR/EFEMP1mut‐2–Flag (mutant 2 codons, CCC, TAT) or EGFR/EFEMP1mut‐9–Flag (mutant 9 codons). (I) Western blotting analysis of phosphorylation of EGFR and downstream signalling substrates

To elucidate the mechanism through which METTL1‐mediated m^7^G tRNA modification upregulates EGFR/EFEMP1 protein expression, we analysed Ribo‐sequencing data using the integrative genomics viewer (IGV). The results indicated that the peaks of translated EGFR/EFEMP1 mRNA were reduced upon METTL1 depletion and increased after rescue (Figure 7A,B). Polyribosome‐RNA‐qPCR was conducted to verify that METTL1 regulated the expression of EGFR/EFEMP1 at the translational level. METTL1 depletion reduced the TE of EGFR/EFEMP1. METTL1 rescue restored TE (Figure 7C,D). Codon frequency analysis revealed more m^7^G tRNA‐decoded codons relative to non‐m^7^G tRNA‐decoded codons encoding the same amino acid (Figure 7E). To establish whether EGFR/EFEMP1 mRNA requires m^7^G tRNA modifications during translation, we generated cDNAs of EGFR/EFEMP1 mutants in which m^7^G tRNA‐decoded codons were replaced by their non‐m^7^G tRNA decoded synonymous counterparts (Figure 7F). Equal amounts of wild‐type or mutant EGFR/EFEMP1 cDNAs were transiently transfected into T24 empty vector control cells or T24 METTL1‐depletied cells. Western blotting analysis indicted that the level of EGFR/EFEMP1 in wild‐type‐transfected T24 control cells was higher than that in cells transfected with the mutant with nine codons replaced but was not higher than in cells transfected with the mutant with two codons (CCC, TAT) replaced. In contrast, there were no significant changes between the wild‐type and mutant EGFR/EFEMP1 in T24 METTL1‐depleted cells (Figure 7G,H). These data suggested that METTL1 upregulates the expression of EGFR/EFEMP1 by affecting the level of m^7^G tRNA. Given that METTL1 depletion suppressed the expression of EGFR/EFEMP1, we sought to determine whether METTL1 regulates EGFR downstream signalling pathways. The Western blot revealed that METTL1 depletion suppressed FAK/Akt signalling (Figure 7I), suggesting that METTL1 promoted tumourigenesis in BC through the EGFR pathway. Zhang et al.^22^ previously found that internal m^7^G facilitates mRNA translation. To investigate whether internal EGFR/EFEMP1 mRNA positions underwent m^7^G modification, we performed a methylated RNA‐immunoprecipitation‐qPCR (MeRIP‐qPCR) assay. We specifically removed cap m^7^G, but not internal m^7^G, using the RppH enzyme (Figure S7A). m^7^G‐modified EGFR/EFEMP1 transcripts were not detected in T24 cells via RT‐qPCR (very low m^7^G IP/input), and no striking differences were observed between T24 control and METTL1 knockout cells (Figure S7B). Taken together, our data suggested that METTL1‐mediated m^7^G tRNA modifications regulate the translation of EGFR/EFEMP1, in turn promoting BC progression via the EGFR pathway.

### 3.7 METTL1 promotes bladder cancer tumourigenesis via the epidermal growth factor receptor pathway

**FIGURE 8:**
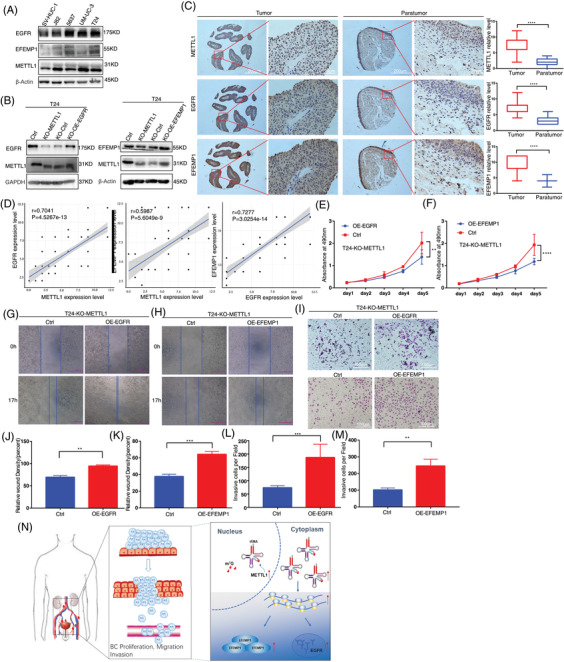
METTL1 promotes bladder cancer (BC) cell tumourigenesis through epidermal growth factor receptor (EGFR) pathway. (A) WB analysis of EGFR/EFEMP1 in SV‐HUC‐1 and BC cell lines. (B) WB analysis of overexpression of EGFR/EFEMP1 in METTL1 knockout T24 cells. (C) Representative immunohistochemistry (IHC) staining images and statistical analysis of METTL1/EGFR/EFEMP1 expression in tumour tissues (*n *= 63) and matched paratumour (*n *= 16) tissues in BC cancer tissue microarray (TMA; ****P < 0.0001). Scale bars: (left panel) 200 μm, (right panel) 20 μm. The *Y*‐axis is the semiquantitative histopathological scoring. (D) Correlation between EGFR/EFEMP1 expression and METTL1 expression in BC tissue microarray. (E,F) The MTT assays showed that the proliferation capacities were significantly enhanced in overexpression of EGFR/EFEMP1 after METTL1 knockout cells (**P < 0.01, ***P < 0.0001). (G,H,J,K) The wound healing assays demonstrated that overexpression of EGFR/EFEMP1 in METTL1 depletion cells promoted cell migration abilities (**P < 0.01, ***P < 0.001). (I,L,M) The transwell invasion assays demonstrated that overexpression of EGFR/EFEMP1 in METTL1 depletion cells promoted cell invasion abilities (**P < 0.01, ***P < 0.001). Scale bars: (I) 100 μm. (N) Summary of the METTL1/EGFR/EFEMP1 signalling pathway

To further confirm whether METTL1 regulated BC tumourigenesis through the EGFR signalling pathway, we detected EGFR/EFEMP1 levels in BC cell lines. The Western blot revealed that METTL1, EGFR and EFEMP1 were upregulated, and a high correlation was observed between the former and EGFR/EFEMP1 in advanced cells (Figure 8A). To further validate this finding, we assessed the protein expression of METTL1/ EGFR/EFEMP1 in BC TMA slides (*n* = 79) via IHC. METTL1, EGFR and EFEMP1 were moderately or highly expressed in the majority of BC samples, while weak or no expression was observed in most paracancerous tissues (Figure 8C). IHC confirmed the positive correlation of EGFR and EFEMP1 expression with that of METTL1 (Figure 8D). Next, we overexpressed EGFR/EFEMP1 in METTL1 knockout T24 cells (Figure 8B). MTT assays revealed that the proliferation ability of METTL1 knockout cells was remarkably increased by overexpression of EGFR/EFEMP1 (Figure 8E,F). In addition, EGFR/EFEMP1 overexpression promoted cell migration and invasion abilities, as indicated by the respective functional assays (Figure 8G–M). We further knocked down EGFR/EFEMP1 in METTL1 overexpression SV‐HUC‐1 cells using siRNAs. WB results confirmed that EGFR siRNA‐1 and EFEMP1 siRNA‐1 had the best interference effect and were selected for following assays (Figures S8A–C). MTS and scratch wound assays showed that either or both EGFR/EFEMP1 knockdown decreased the proliferation and migration abilities of METTL1 overexpression SV‐HUC‐1 cells (Figure S8D–F). Those results suggested that knockdown either or both EGFR/EFEMP1 abolished the effect of METTL1 overexpression in SV‐HUC‐1 cells. Taken together, METTL1 promoted BC cell tumourigenesis via the EGFR pathway.

## 4 DISCUSSION

Dynamic RNA modifications have emerged as novel mechanisms regulating gene expression, of which tRNA modifications are suggested to coordinate translation by controlling tRNA stability, folding and function.^11^, ^12^, ^13^, ^14^, ^15^ Recent studies have revealed that the dysregulation of tRNA m^7^G methyltransferase METTL1 is associated with a malignant phenotype in a number of disorders, including cancer.^20^, ^25^, ^26^ During the submission of this work, three published papers also reported that METTL1 is overexpressed in cancers such as AML, intrahepatic cholangiocarcinoma (ICC), lung cancer and is related with poor patient survival ^34^, ^35^, ^36^. These studies implicate an essential role of METTL1 in the cancer development. However, its exact role and detailed regulatory mechanism in BC progression are currently unknown. In this study, we demonstrated that the level of METTL1 was highly upregulated in BC and correlated with poor prognosis. Further, METTL1 promoted the proliferation, migration and invasion of BC cells through the regulation of the EGFR/EFEMP1 translation. The current results highlight the oncogenic role of METTL1 and the relevance of the METTL1‐m^7^G‐EGFR/EFEMP1 axis in the BC development, indicating that METTL1 is for improved BC treatment as potential therapeutic target.

Through loss‐ and gain‐of‐function analyses, we demonstrated that METTL1 promotes BC cell proliferation, migration, and invasion both in vitro and in vivo. In particular, we established a zebrafish xenograft cancer model for the visualization of tumour progression. Most organ systems are functionally similar between zebrafish and humans, and 82% of disease‐related human proteins have orthologs in zebrafish.^37^ Therefore, the zebrafish embryo is an ideal model for studying tumour formation and progression.^38^, ^39^, ^40^ In our study, the proliferation and metastatic behaviour of T24 cells was clearly observed in model zebrafish. Xenograft assays in mice further confirmed that METTL1 depletion suppressed tumour progression in vivo. Thus, we concluded that METTL1 serves an oncogenic role in BC.

Considering that METTL1 has been confirmed as a tRNA m^7^G methyltransferase, Lin et al.^30^ previously developed an antibody‐independent, TRAC‐seq approach in order to explore the mammalian m^7^G tRNA methylome at single‐nucleotide resolution, in turn identifying a specific subset of 22 tRNAs that undergo m^7^G modification in mouse embryonic stem cells (mESCs). Marchand et al.^41^ employed AlkAniline‐Seq to validate 17 m^7^G‐tRNAs in human HCT116 cells. Orellana et al. identified a subset of 25 m^7^G modified‐tRNAs in human GBM cell line LNZ308 by TRAC‐seq.^36^ In the current study, we identified 20 m^7^G‐tRNAs in T24 cells using TRAC‐seq, which were consistent with those reported by Lin et al., except for tRNA‐Cys‐GCA and tRNA‐Gly‐ACC. These results indicate that m^7^G‐tRNAs are tissue‐and cell‐type specific, suggesting a cellular context‐dependent function. Meanwhile, we demonstrated that METTL1‐mediated m^7^G modification affected tRNA abundance and could selectively regulate the translation of transcripts with a higher frequency of m^7^G‐tRNA decoding codons.

Indeed, as an m^7^G methyltransferase, the molecular mechanism of METTL1 is highly complex and multifaceted. Apart from METTL1's known function in m^7^G tRNA modification, recent studies have revealed that it can install a subset of internal m^7^G sites in human mRNA and miRNA, in turn increasing TE and miRNA processing, respectively.^21^, ^22^ Previous studies have reported that METTL1 modulates the miR‐149‐3p/S100A4/p53 axis, let‐7e miRNA/HMGA2 axis, and the PTEN pathway in colon cancer as well as hepatocellular carcinoma.^20^, ^25^, ^42^ More recently, Orellana et al.^36^ and Dai et al.^34^ demonstrated that m^7^G modification by the METTL1/WDR4 complex promotes a subset of tRNAs stabilization and increases translation of cell cycle promoting mRNAs (CCNA2, CCND2, CDK6, CDK8, and so on) and pro‐oncogenic mRNAs (EGFR, KDM1a, etc.), resulting in cell transformation and cancer progression. Ma et al.^35^ revealed that knockdown METTL1 reduces the translation of mRNAs which have higher frequencies of m^7^G tRNA codons, causing decreased tumourigenic abilities in lung cancer. Through multi‐omics (transcriptome, translatome, proteome and m^7^G tRNA methylome) analysis, we sought to determine potential targets of METTL1 in BC. Our results indicated that DEGs under METTL1 knockout and overexpression were enriched in cancer‐related pathways, such as the PI3K‐Akt, MAPK and BC signalling. Importantly, proteomic analysis and *de novo* protein assays indicated that METTL1‐mediated m^7^G tRNA modification upregulated EGFR/EFEMP1 protein expression. We further validated the levels of translated mRNA and the frequency of m^7^G tRNA‐decoded codons for EGFR/EFEMP1. In addition, we performed an MeRIP‐qPCR assay to rule out internal m^7^G sites in EGFR/EFEMP1.Taken together, these analyses revealed that the METTL1‐mediated m^7^G tRNA modification regulated EGFR/EFEMP1 translation.

The EGFR signalling pathway is a central driver of tumourigenesis and a major drug target in various solid malignancies, including lung, breast, colon and bladder tumours.^43^, ^44^, ^45^, ^46^, ^47^, ^48^, ^49^ Aberrant activation and expression of EGFR have been frequently reported in BC.^50^, ^51^, ^52^, ^53^, ^54^ These promote the proliferation, motility and invasive capacity of BC cells. EFEMP1 (Fibulin‐3, FBLN3) is an EGF‐containing fibulin‐like extracellular matrix protein, which has been reported to act as both a tumour promoter and suppressor, depending on the cancer type.^33^, ^55^, ^56^, ^57^, ^58^ EFEMP1 was shown to suppress the glioma growth by modulating the tumour microenvironment.^58^ Conversely, EFEMP1 enhanced pancreatic carcinoma cell growth by binding to EGFR to activate MAPK and Akt pathways.^33^ A recent study found that EFEMP1 acts as a pro‐invasive factor in BC, mediating its effects through the modulation of IGFBP5 expression.^55^ In this study, we showed that METTL1 promoted BC progression by modulating EGFR/EFEMP1 translation to activate EGFR signalling. More importantly, EGFR/EFEMP1 overexpression reversed the suppressive role of METTL1 depletion on BC cell proliferation, migration and invasion in vitro. Taken together, we present a comprehensive analysis exploring the relationship between tRNA abundance, codon usage and EGFR/EFEMP1 translation, to uncover the molecular mechanism underlying METTL1's oncogenic function in BC, adding to a limited body of knowledge regarding the significance of METTL1 in BC.

Overall, the current study is the first to provide robust in vitro and in vivo evidence supporting the oncogenic role of METTL1‐mediated m^7^G tRNA modification in BC. Of note, we clarified the underlying molecular mechanisms of METTL1‐mediated m^7^G tRNA modification, namely its regulation of EGFR/EFEMP1 translation, thus providing a theoretical basis for the development of novel targeted therapies against BC.

## CONFLICT OF INTEREST

The authors declare that there is no conflict of interest that could be perceived as prejudicing the impartiality of the research reported.

## Supporting information



Supporting information
