# Supplementary material for: METTL1‐m7G‐EGFR/EFEMP1 axis promotes the bladder cancer development
Source: Clin Transl Med. 2021 Dec 22;11(12):e675. doi: 10.1002/ctm2.675 (PMC8694502; doi:10.1002/ctm2.675)
Supplement: Supplementary file 1 — Supporting information [file CTM2-11-e675-s001.docx]

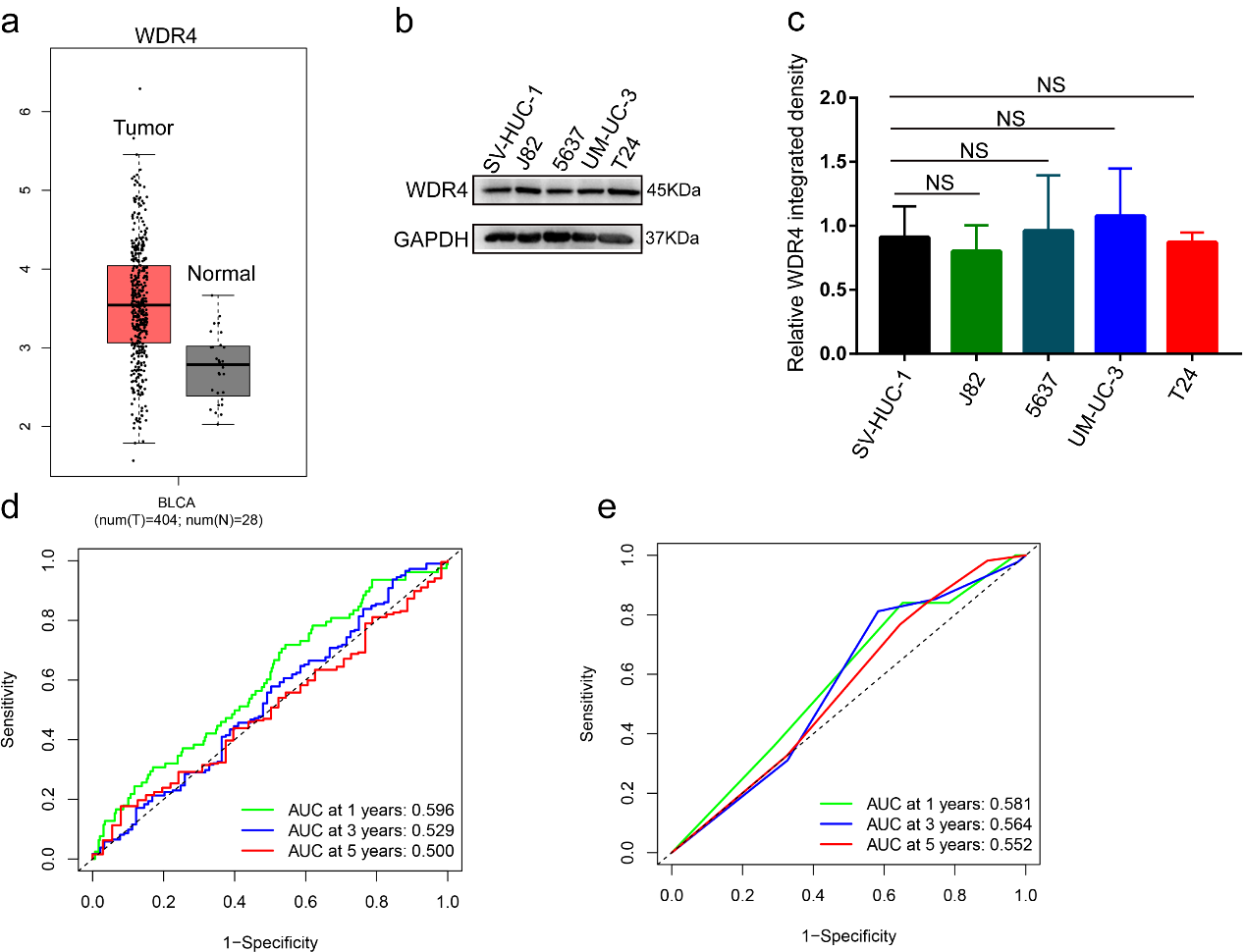


**Supplementary Figure 1. The expression of WDR4 in tumor and normal tissue or cells and** **ROC curve analysis of METTL1. a,** Comparison of WDR4 mRNA expression between tumor and normal tissue in TCGA dataset (n = 404 in tumor and n = 28 in normal, P>0.05). **b,** western blot analysis of WDR4 expression in normal and BC cell lines. **c,** the quantification of western blot data (*P* > 0.05). **d**, the ROC curve analysis of METTL1 in TCGA data. **e**, the ROC curve analysis of METTL1 in BC tissue microarray.


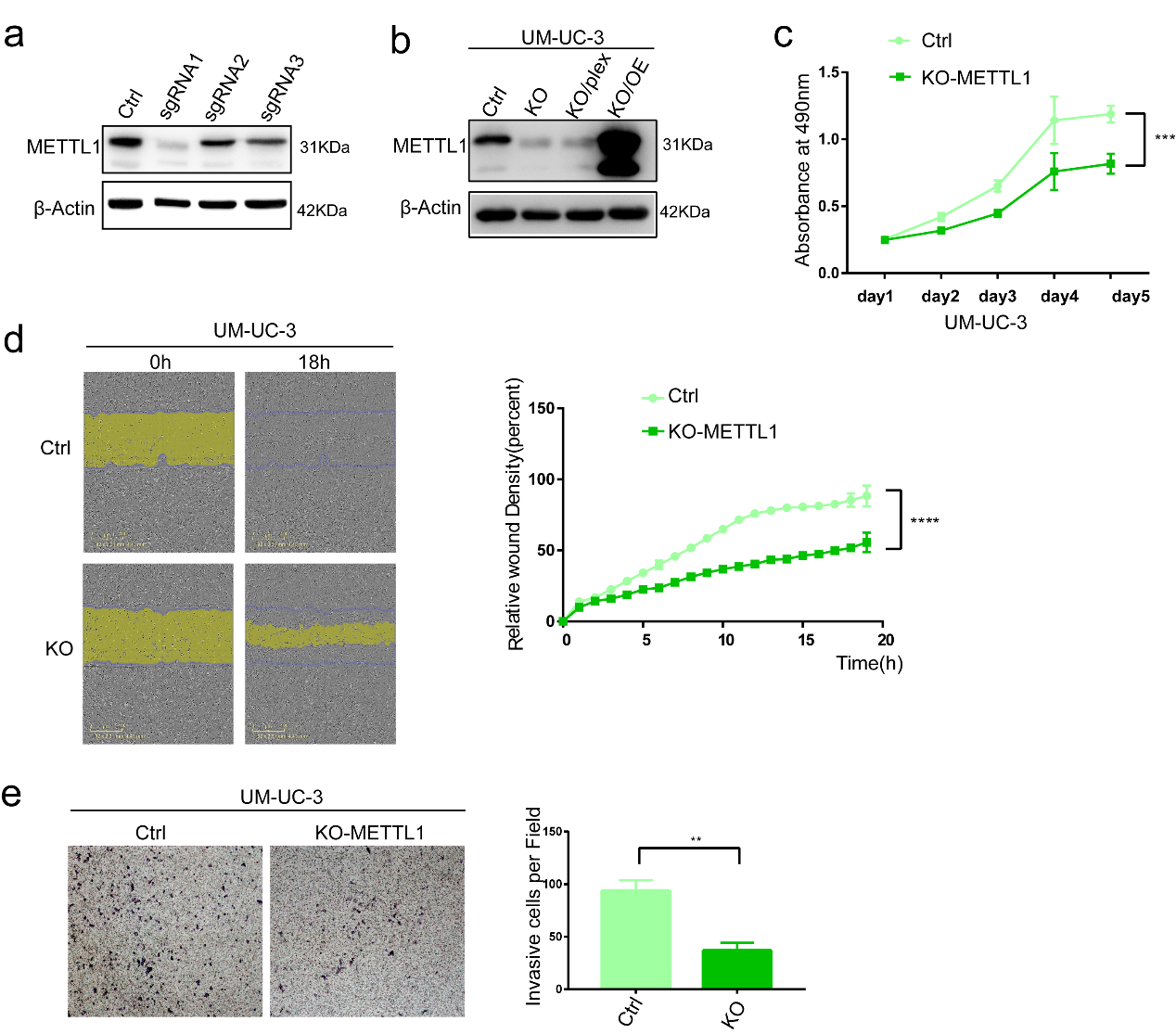


**Supplementary Figure 2. METTL1 promotes tumorigenesis in vitro. a,** METTL1 knockout BC cells were generated using CRSPR/Cas9 technology. **b,** western blot analysis of METTL1 expression in METTL1 stable knockout or rescue cells. **c,** MTS assay showed that METTL1 knockout could reduce the viability of UM-UC-3 cells (****P* < 0.001). **d,** Cell scratch migration assay revealed that the migration capacity was impaired by METTL1 downregulation (*****P* < 0.0001). **e,** Transwell invasion assays showed that METTL1 knockout could decrease the invasive abilities of UM-UC-3 cells (***P* < 0.01).


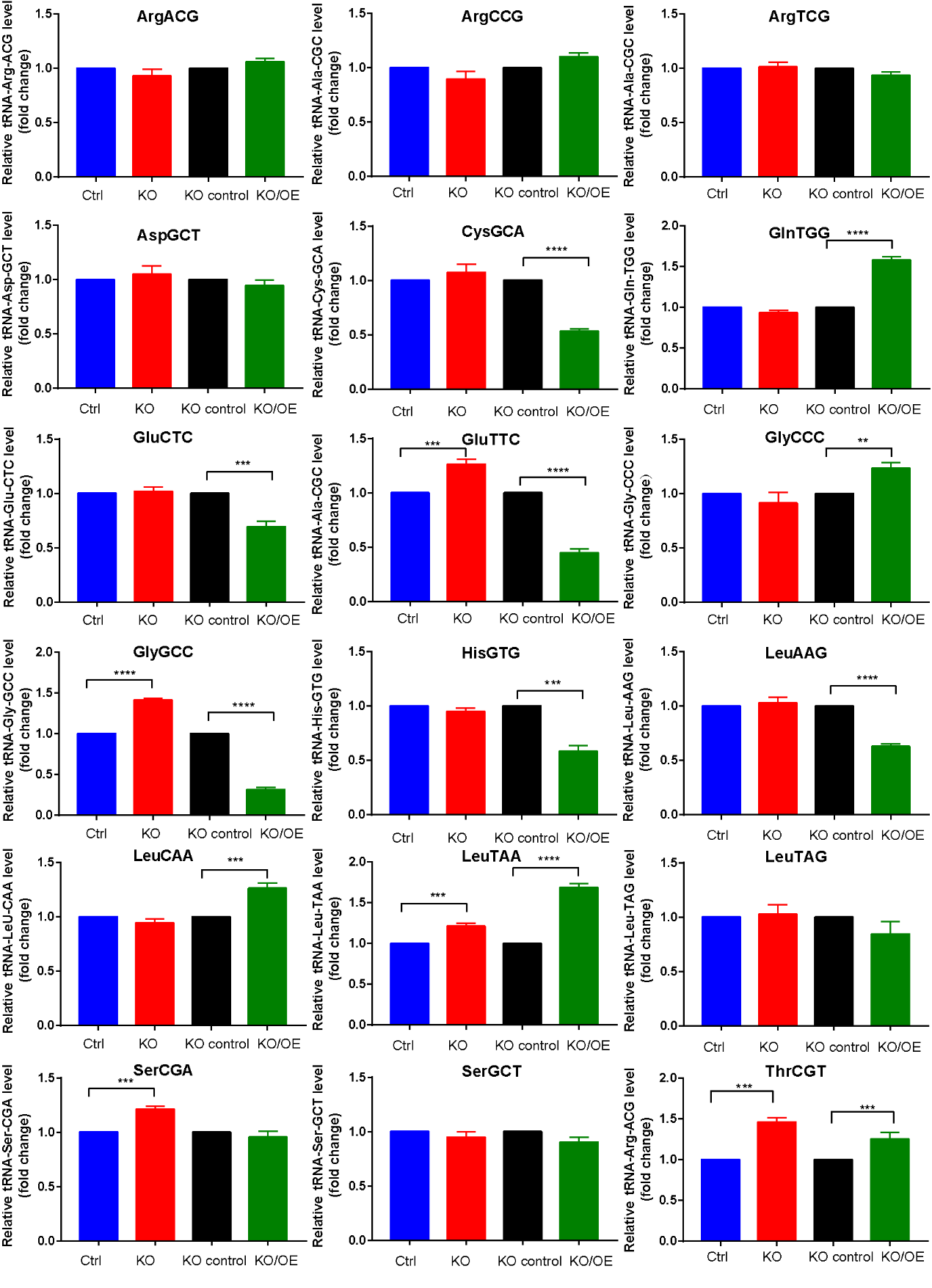


**Supplementary Figure 3. METTL1 mediated m^7^G tRNA modification did not affect the level of non-m^7^G tRNAs.** analysis of abundance of tRNAs without m^7^G modification using RT-qPCR (***P*<0.01, ****P* < 0.001, *****P* < 0.0001).


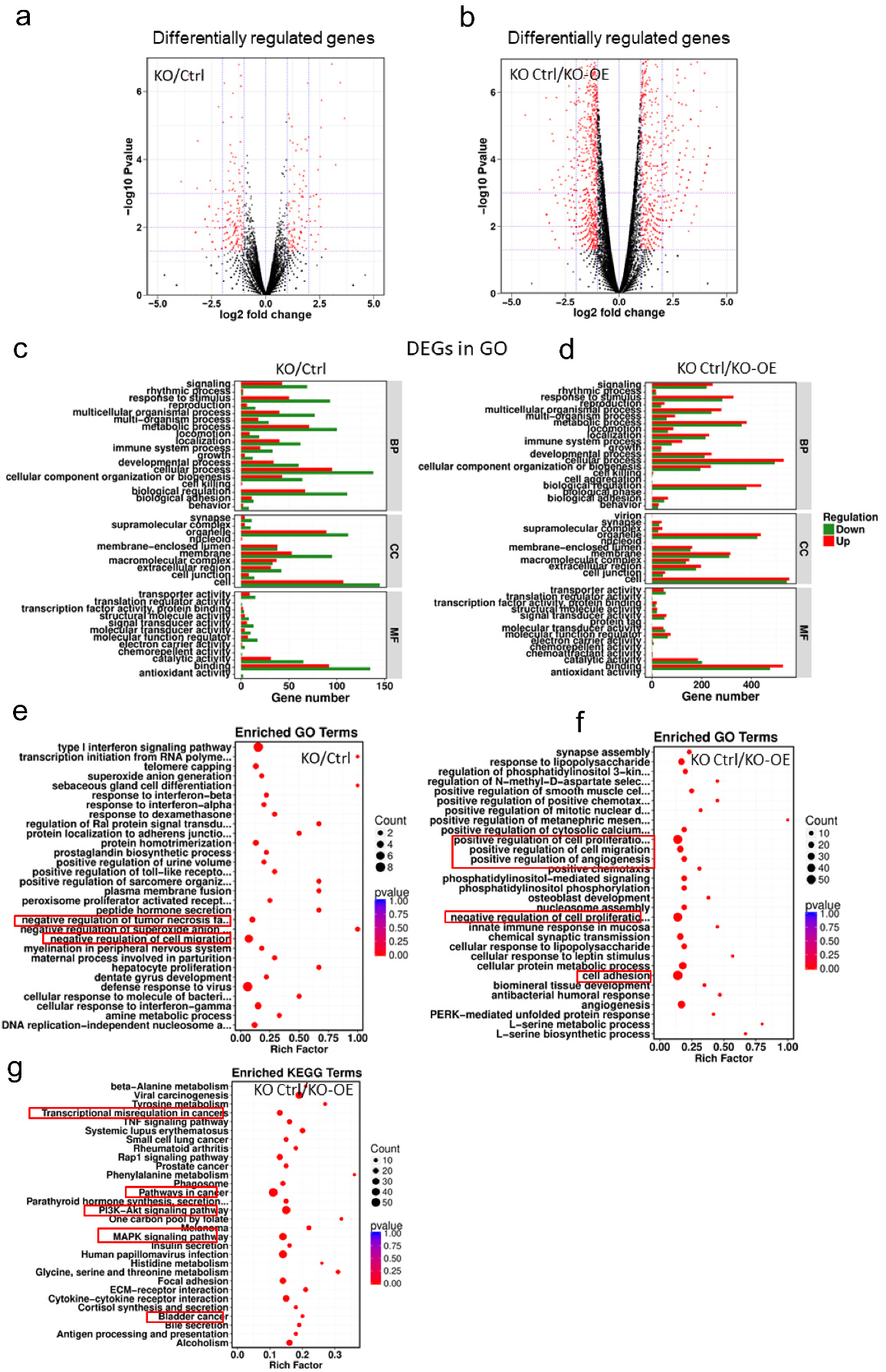


**Supplementary Figure 4. Transcriptome analysis in METTL1 KO and METTL1 rescued groups compared with control groups by RNA-Seq.** **a,** The volcano plot of DEGs between METTL1 KO group and empty vector control group. **b,** the volcano plot of DEGs between METTL1 rescued group and empty vector control group. **c, d,** GO analysis of DEGs from RNA-seq data. **e, f,** GO analysis of DEGs from RNA-seq data in Biological Process. **g,** KEGG pathway enrichment analysis of DEGs.


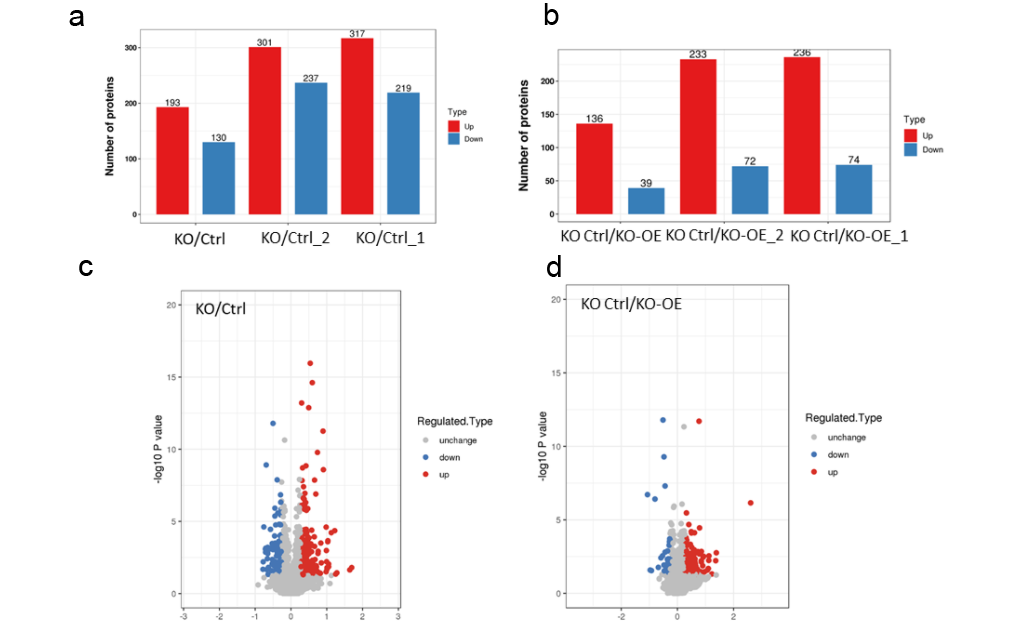


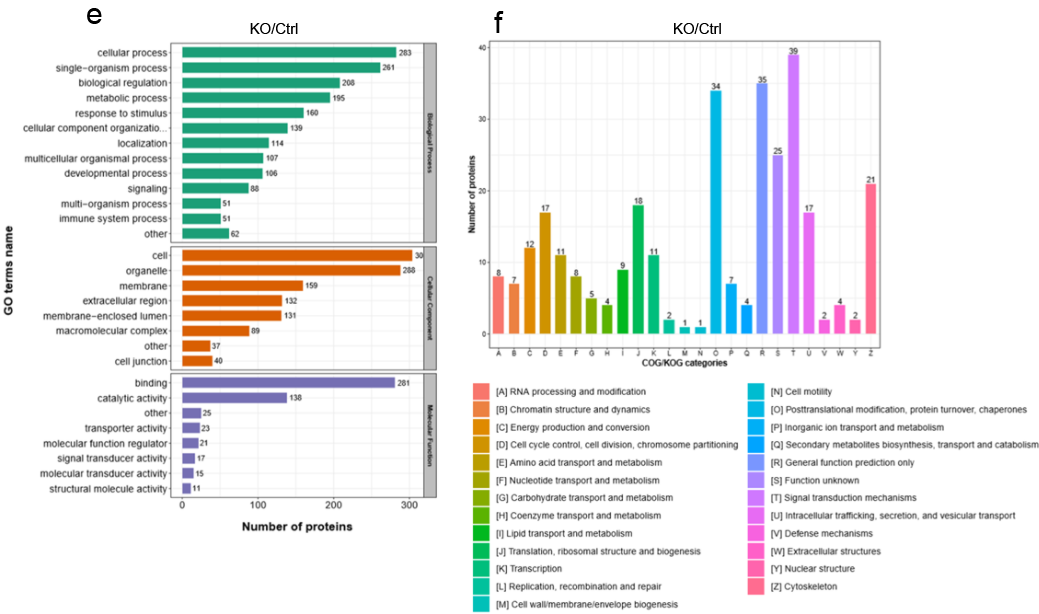


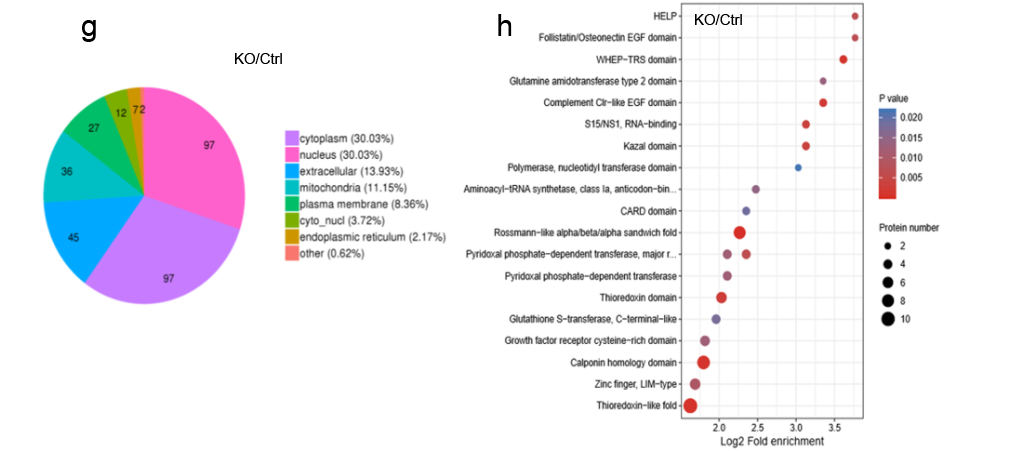


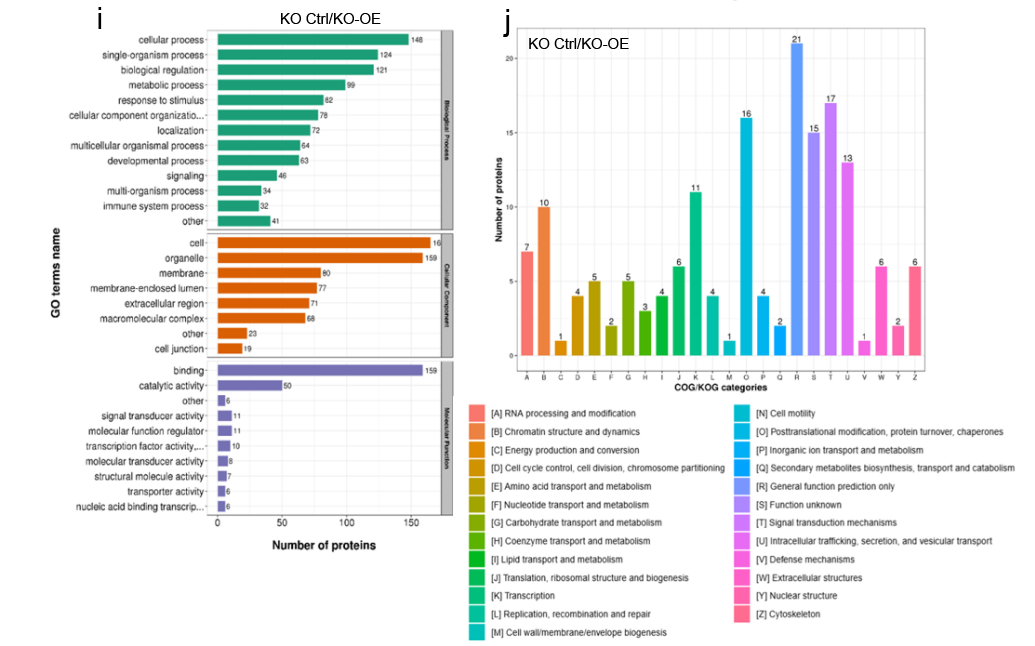


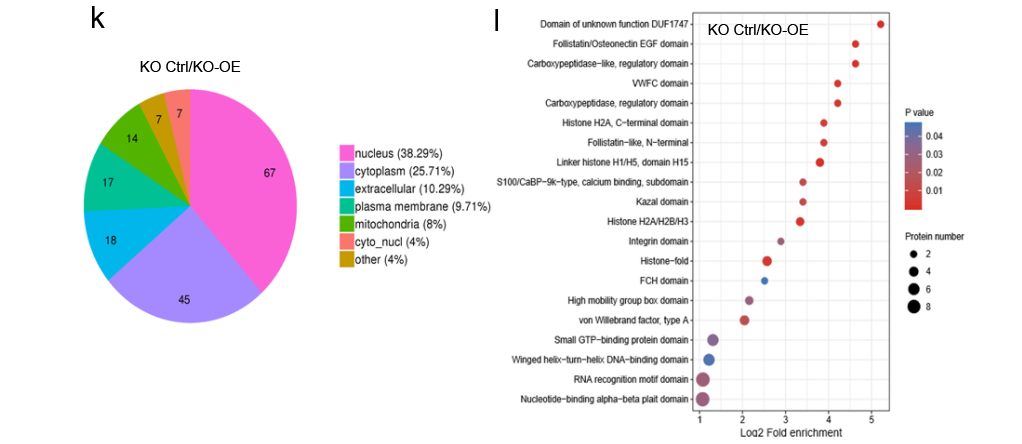


**Supplementary Figure 5. Proteome analysis in METTL1 KO and METTL1 rescued groups compared with control groups by protein-Seq.** **a, b,** Histograms showing the numbers of differentially expressed proteins (DEPs) in various comparative groups. **c, d,** Volcano plot of DEPs. **e, I,** GO analysis of DEPs from protein-seq data. **f, j,** COG analysis of DEPs from protein-seq data. **g, k,** subcellular localization analysis of DEPs. **h, l,** Protein domain analysis of DEPs.


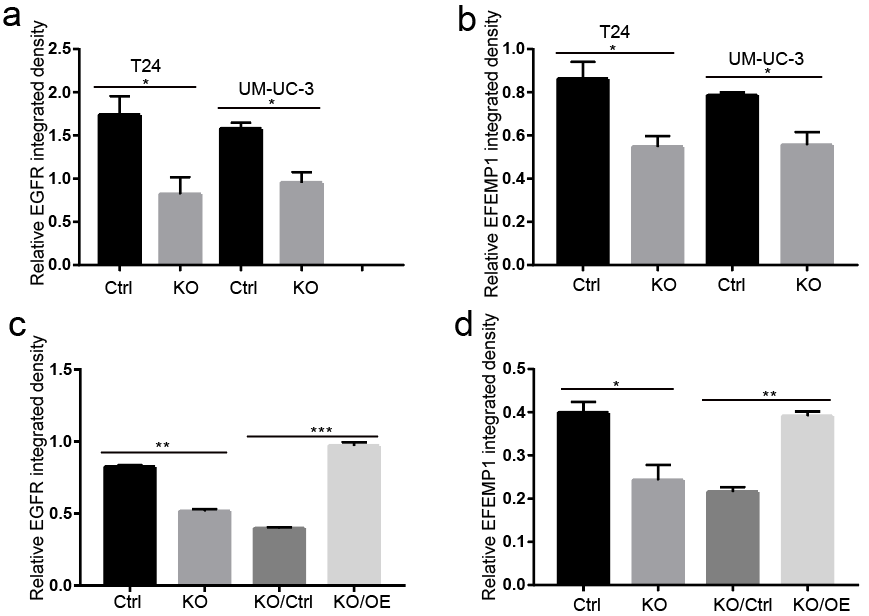


**Supplementary Figure 6. Statistical analysis of the protein expression levels. a, b** Statistical analysis protein expression of EGFR and EFEMP1 upon METTL1 KO in T24 and UM-UC-3 cells in western blotting. **c, d** Statistical analysis of protein expression of EGFR and EFEMP1 upon METTL1 KO or overexpression METTL1 after knockout METTL1 in T24 cells. (*P<0.05, **P<0.01, ***P<0.001).


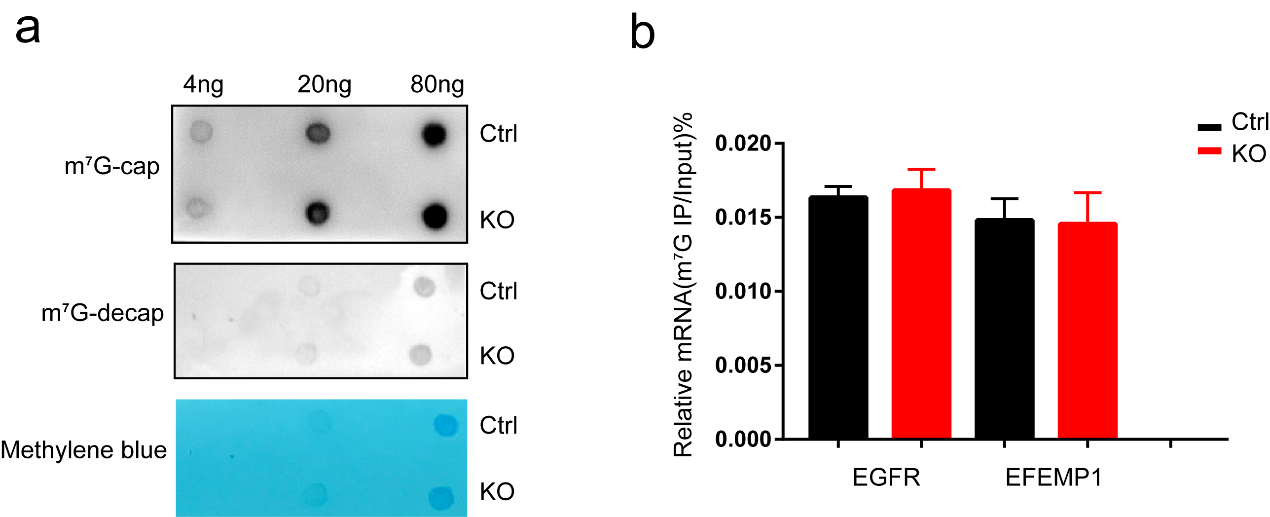


**Supplementary Figure 7.** **The internal EGFR/EFEMP1 mRNA levels were determined using methylated RNA-immunoprecipitation-qPCR. a**, Dot blot analysis of m^7^G level. Methylene blue staining served as a loading control (lower panel) **b,** methylated RNA-immunoprecipitation-qPCR analysis of the internal EGFR/EFEMP1 mRNA levels.


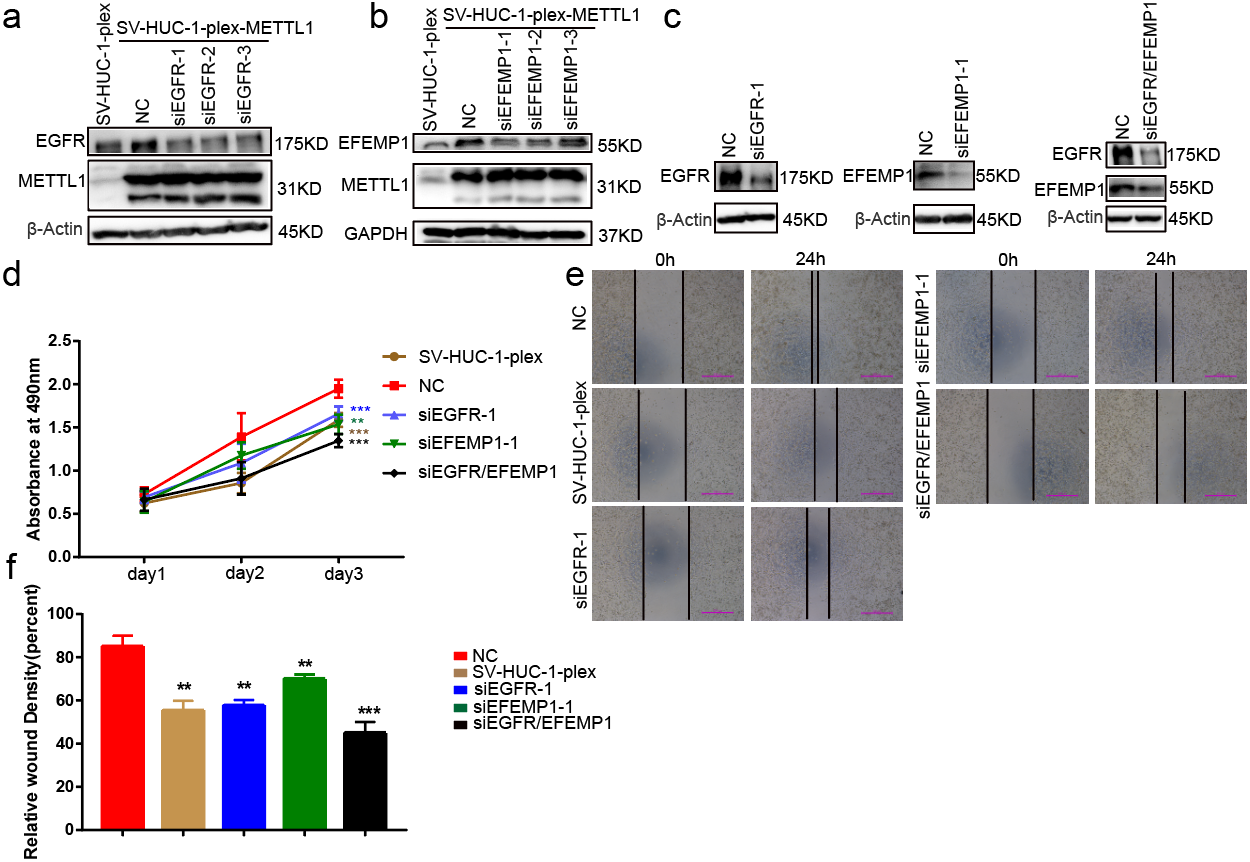


**Supplementary Figure 8. EGFR/EFEMP1 knockdown decreased the proliferation and migration abilities of** **METTL1 overexpression SV-HUC-1 cells. a-c**, western blot analysis EGFR/EFEMP1 expression in METTL1 overexpression SV-HUC-1 cells using siRNAs. **d-f,** MTS and scratch wound assays showed that either or both EGFR/EFEMP1 knockdown decreased the proliferation and migration abilities of METTL1 overexpression SV-HUC-1 cells (**P<0.01, ***P<0.001).

**Supplementary Table 1**. Baseline information of 130 patients with BC

| Characteristic Number (percentage) |
| --- |
| Gender  Female 28 (21.5%)  Male 102 (78.5%)  Age  <60 41(31.5%)  ≥60 89(68.5%)  Stage  NMIBC (Ta/T1/Tis) 64(49.2%)  MIBC (T2-T4) 66(50.8%) |

**Supplementary Table 2**. Sequences of sgRNA, siRNAs and primers

| METTL1-sgRNA1-oligo1 | CACCGCGGTAGTAGCGCTTCTGGGG |
| --- | --- |
| METTL1-sgRNA1-oligo2 | AAACCCCCAGAAGCGCTACTACCGC |
| METTL1-sgRNA2-oligo1 | CACCGTTGGGGTGAGTTGGGATCGG |
| METTL1-sgRNA2-oligo2 | AAACCCGATCCCAACTCACCCCAAC |
| METTL1-sgRNA3-oligo1 | CACCGCACGCTGCGCTAGTGAGGAG |
| METTL1-sgRNA3-oligo2 | AAACCTCCTCACTAGCGCAGCGTGC |
| Plex-METTL1-F | CGCGGATCCATGGCAGCCGAGACTCGGAACG |
| Plex-METTL1-R | AAGGAAAAAAGCGGCCGCTCAGTGACCAGGCAGGCTGGTT |
| Plex-EGFR-F | CGCGGATCCATGCGACCCTCCGGGACG |
| Plex-EGFR-R | AAGGAAAAAAGCGGCCGCTCATGCTCCAATAAATTCACTGC |
| Plex-EFEMP1-F | CGCGGATCCATGTTGAAAGCCCTTTTCCTAA |
| Plex-EFEMP1-R | AAGGAAAAAAGCGGCCGCCTAAAATGAAAATGGCCCCACT |
| si-EGFR-1 | CACAGUGGAGCGAAUUCCUUUGGAA |
| si-EGFR-2 | GGAUCCCAGAAGGUGAGAAAGUUAA |
| si-EGFR-3 | CACCGUGGCUUGCAUUGAUAGAAAU |
| si-EFEMP1-1 | CAGACGCUUGTAAAGGUGGAAUGAA |
| si-EFEMP1-2 | CAGACUGGCCGAAAUAACUUUGUCA |
| si-EFEMP1-3 | CGGGAGGAUGAAAUGUGUUGGAAUU |

**Supplementary Table 3.** Key resources table

| REAGENT or RESOURCE | SOURCE | IDENTIFIER |
| --- | --- | --- |
| Antibodies |  |  |
| Rabbit polyclonal anti-Mettl1 | Proteintech | Cat# 14994-1-AP |
| Rabbit monoclonal anti-WDR4 | Abcam | Cat# ab169526 |
| Mouse monoclonal 7-methylguanosine (m^7^G) | MBL International | Cat# RN017M |
| Mouse polyclonal anti-EGFR | Proteintech | Cat# 66455-1-1g |
| Rabbit monoclonal anti-EGFR | Cell Signaling Technology | Cat# 4267 |
| Mouse monoclonal anti- Phospho-EGF Receptor (Tyr1068) | Cell Signaling Technology | Cat# 2236S |
| Rabbit anti-Phospho-Akt (Ser473) | Cell Signaling Technology | Cat# 9271S |
| Rabbit anti- Akt | Cell Signaling Technology | Cat# 9272S |
| Rabbit anti- Phospho-FAK (Tyr576/577) | Cell Signaling Technology | Cat# 3281S |
| Rabbit anti- Phospho-FAK (Tyr397) | Cell Signaling Technology | Cat# 3283S |
| Rabbit anti- FAK | Cell Signaling Technology | Cat# 3285S |
| Rabbit polyclonal anti-beta-Acin | Cell Signaling Technology | Cat# 4970s |
| Rabbit polyclonal anti-EFEMP1 | Abcam | Cat# ab106429 |
| Rabbit polyclonal anti-EFEMP1 | Abcam | Cat# ab256457 |
| Anti-rabbit IgG HRP-lined | Cell Signaling Technology | Cat# 7074s |
| Anti-mouse IgG HRP-lined | Cell Signaling Technology | Cat# 7076s |
| Alexa Fluor^TM^ 568 donkey anti-mouse IgG(H+L) | Invitrogen | Cat# A10037 |
| Alexa Fluor^TM^ 488 donkey anti-rabbit IgG(H+L) | Invitrogen | Cat# A21206 |
| Alexa Fluor^TM^ 568 donkey anti-rabbit IgG(H+L) | Invitrogen | Cat#A10042 |

**Supplementary Table 4.** RT-qPCR primer sequences

| Name | Species | sequences（5' to 3'） |
| --- | --- | --- |
| β-Actin | Homo sapiens | Forward: CCTTGCACATGCCGGAG |
|  |  | Reverse: GCACAGAGCCTCGCCTT |
| METTL1 | Homo sapiens | Forward: GGCAACGTGCTCACTCCAA |
|  |  | Reverse:CACAGCCTATGTCTGCAAACT |
| EGFR | Homo sapiens | Forward: GTCGGGCTCTGGAGGAAAA |
|  |  | Reverse: ATTCCCAAGGACCACCTCAC |
| EFEMP1 | Homo sapiens | Forward:GTCACAGGACACCGAAGAAAC |
|  |  | Reverse:TTGCATTGCTGTCTCACAGGA |
| tRNA-Tyr-GTA | Homo sapiens | Forward: CCTTCGATAGCTCAGCTGGT |
|  |  | Reverse: TCCTTCGAGCCGGAATCGAA |
| tRNA-Val-CAC | Homo sapiens | Forward: GTTTCCGTAGTGTAGTGGTT |
|  |  | Reverse: TGTTTCCGCCCGGTTTCGAA |
| tRNA-Asn-GTT | Homo sapiens | Forward: GTCTCTGTGGCGCAATCGGT |
|  |  | Reverse: CGTCCCTGGGTGGGCTCGAA |
| tRNA-Trp-CCA | Homo sapiens | Forward: GACCTCGTGGCGCAACGGTA |
|  |  | Reverse: TGACCCCGACGTGATTTGAA |
| tRNA-Val-AAC | Homo sapiens | Forward: GTTTCCGTAGTGTAGTGGTT |
|  |  | Reverse: TGTTTCTGCCCGGTTTCGAA |
| tRNA-Val-TAC | Homo sapiens | Forward: GGTTCCATAGTGTAGTGGTT |
|  |  | Reverse: TGGTTCCACTGGGGCTCGAA |
| tRNA-Ala-AGC | Homo sapiens | Forward: GGGGAATTAGCTCAAGTGGT |
|  |  | Reverse: TGGAGAATGTGGGCATCGAT |
| tRNA-Ala-CGC | Homo sapiens | Forward: GGGGATGTAGCTCAGTGGTA |
|  |  | Reverse: TGGAGATGCCGGGGATCGAA |
| tRNA-Ile-AAT | Homo sapiens | Forward: GGTTAGCTCAGTCGGCTAGA |
|  |  | Reverse: TGGCCCGTACGGGGATCGAA |
| tRNA-Ile-TAT | Homo sapiens | Forward: GCTCCAGTGGCGCAATCGGT |
|  |  | Reverse: TGCTCCAGGTGAGGCTCGAA |
| tRNA-Lys-CTT | Homo sapiens | Forward: GCCCGGCTAGCTCAGTCGGT |
|  |  | Reverse: CGCCCAACGTGGGGCTCGAA |
| tRNA-Lys-TTT | Homo sapiens | Forward: GGATAGCTCAGTCGGTAGAG |
|  |  | Reverse: GAACAGGGACTTGAACCCTG |
| tRNA-Met | Homo sapiens | Forward: GCCCTCTTAGCGCAGCGG |
|  |  | Reverse: TGCCCTCTCTGAGGCTCGAA |
| tRNA-Phe-GAA | Homo sapiens | Forward: GCCGAAATAGCTCAGTTGGG |
|  |  | Reverse: TGCCGAAACCCGGGATCGAA |
| tRNA-Thr-TGT | Homo sapiens | Forward: GGCTCCATAGCTCAGGGGTT |
|  |  | Reverse: AGGCCCCAGCGAGATTTGAA |
| tRNA-Thr-AGT | Homo sapiens | Forward: GGCTCCGUGGCTTAGCTGGT |
|  |  | Reverse: AGGCCCCGCTGGGATTCGAA |
| tRNA-Thr-CGT | Homo sapiens | Forward: GGCTCTGTGGCTTAGTTGGC |
|  |  | Reverse: AGGCCCCGCTGGGATTCGAA |
| tRNA-His-GTG | Homo sapiens | Forward: GCCGTGATCGTATAGTGGTT |
|  |  | Reverse: TGCCGTGACTCGGATTCGAA |
| tRNA-Leu-AAG | Homo sapiens | Forward: GGTAGCGTGGCCGAGCGGTC |
|  |  | Reverse: TGGCAGCGGTGGGATTCGAA |
| tRNA-Leu-CAA | Homo sapiens | Forward: GTCAGGATGGCCGAGTGGTC |
|  |  | Reverse: TGTCAGAAGTGGGATTCGAA |
| tRNA-Leu-TAA | Homo sapiens | Forward: ATGGCCGAGTGGTTAAGGCG |
|  |  | Reverse: TACCAGGAGTGGGGTTCGAA |
| tRNA-Leu-TAG | Homo sapiens | Forward: GGTAGCGTGGCCGAGTGGTC |
|  |  | Reverse: TGGCAGCGGTGGGATTCGAA |
| tRNA-Ser-AGA | Homo sapiens | Forward: GTAGTCGTGGCCGAGTGGTT |
|  |  | Reverse: CGTAGTCGGCAGGATTCGAA |
| tRNA-Ser-CGA | Homo sapiens | Forward: GCTGTGATGGCCGAGTGGTT |
|  |  | Reverse:CGCTGTGAGCAGGATTTGA |
| tRNA-Ser-GCT | Homo sapiens | Forward:GACGAGGTGGCCGAGTGGTT |
|  |  | Reverse: CGACGAGGATGGGATTCGAA |
| tRNA-Gln-TTG | Homo sapiens | Forward: GGCCCCATGGTGTAATGGTT |
|  |  | Reverse: AGGTCCCACCGAGATTTGAA |
| tRNA-Asp-GTC | Homo sapiens | Forward: TCCTCGTTAGTATAGTGGTG |
|  |  | Reverse: CTCCCCGTCGGGGAATCGAA |
| tRNA-Glu-CTC | Homo sapiens | Forward: TCCCTGGTGGTCTAGTGGTT |
|  |  | Reverse: TTCCCTGACCGGGAATCGAA |
| tRNA-Glu-TTC | Homo sapiens | Forward: TCCCATATGGTCTAGCGGTT |
|  |  | Reverse: TTCCCATACCGGGAGTCGAA |
| tRNA-Gly-CCC | Homo sapiens | Forward: GCATTGGTGGTTCAGTGGTA |
|  |  | Reverse: TGCATTGGCCGGGAATTGAA |
| tRNA-Gly-GCC | Homo sapiens | Forward: GCATGGGTGGTTCAGTGGTA |
|  |  | Reverse: TGCATGGGCCGGGAATCGAA |
| tRNA-Gly-TCC | Homo sapiens | Forward: GCGTTGGTGGUATAGTGGTT |
|  |  | Reverse: TGCGTTGGCCGGGAATCGAA |
| tRNA-Arg-TCT | Homo sapiens | Forward:CGACTCTGGTGGGACTCGAAC |
|  |  | Reverse: CGACTCTGGTGGGACTCGAAC |
| tRNA-Pro-AGG，TGG，CGG | Homo sapiens | Forward: GGCTCGTTGGTCTAGGGGTAT |
|  |  | Reverse: GGGCTCGTCCGGGATTTGAAC |
| U6 | Homo sapiens | Forward: CGCTTCGGCAGCACATATAC |
|  |  | Reverse: TTCACGAATTTGCGTGTCAT |
| tRNA-Cys-GCA | Homo sapiens | Forward: GGGGGTATAGCTCAGGGGTA |
|  |  | Reverse: AGGGGGCACCTGGATTTGAA |
| tRNA-Gln-CTG | Homo sapiens | Forward: GGTTCCATGGTGTAATGGT |
|  |  | Reverse: AGGTTCCACCGAGATTTGAA |
